# Performance Evaluation and Error Mitigation of Ultrasonic Indoor Positioning: An ESP32-Based IMU-ESKF Architecture

**DOI:** 10.3390/s26134090

**Published:** 2026-06-27

**Authors:** Dongze Wang, Mohammed Faeik Ruzaij Al-Okby, Sadegh Refaeiabdolhosseinzadehneishabouri, Mohammed Ali Tlili, Kerstin Thurow

**Affiliations:** 1 Center for Life Science Automation (Celisca), University of Rostock, 18119 Rostock, Germany; wang.dongze@outlook.com (D.W.); sadegh.refaeiabdolhosseinzadehneishabouri@uni-rostock.de (S.R.); mohamed.tlili@uni-rostock.de (M.A.T.); kerstin.thurow@celisca.de (K.T.); 2 Technical Institute of Babylon, Al-Furat Al-Awsat Technical University (ATU), Kufa 54003, Iraq

**Keywords:** ultrasonic indoor positioning, ESP32-S3, inertial measurement unit, error-state Kalman filter, non-line of sight, automated guided vehicle, robot arm, sensor fusion, Marvelmind, ICM-20948

## Abstract

Reliable indoor localization is required for automated guided vehicles (AGVs), robot validation, and industrial digital-twin applications, but ultrasonic positioning can degrade sharply when acoustic visibility changes. This paper evaluates Marvelmind Super-Beacon localization in controlled laboratory experiments involving both AGV tracking and UR10 robot-arm positioning. The non-inverse architecture (NIA) and inverse architecture (IA) configurations are included as parallel validation scenarios to assess the robustness of the proposed mitigation framework across different Marvelmind deployment modes. The baseline analysis identifies the dominant acoustic failure modes, including multipath-induced scatter, crossover-zone handover jumps, update-rate degradation, complete non-line-of-sight (NLoS) outages, and height-dependent 3D jitter. To mitigate these effects, an embedded ultrasonic–inertial pipeline is implemented on an ESP32-S3-WROOM-1 module. The system combines UART packet validation, interrupt-driven ICM-20948 inertial acquisition at 500 Hz, sliding-window kinematic outlier rejection, and a 15-state error-state Kalman filter (ESKF). The embedded estimator logic is designed to maintain motion continuity during intermittent or corrupted acoustic positioning while reintroducing validated ultrasonic absolute corrections. Using recorded AGV and UR10 datasets, mitigation performance was quantitatively assessed through a firmware-consistent replay of the recorded measurements, using the same gating, inertial propagation, and measurement-update logic as the real-time ESP32-S3 implementation. Across ten trials per configuration, the replay-based trial-mean RMSE in the 2D AGV scenarios decreased from 101.2–104.1 mm for raw ultrasonic data to 47.2–48.7 mm after fusion, while peak failure-interval errors were reduced by 64.2–65.7%. In the 3D UR10 scenarios, replay-based trial-mean RMSE decreased from 157.6–158.4 mm to 80.2–80.5 mm, and peak height-sensitive 3D errors were reduced by 58.8–60.0%. The results demonstrate the feasibility of embedded ultrasonic–inertial robustness enhancement for localization in controlled laboratory AGV and robot-arm scenarios. While the proposed approach shows promising performance under the investigated conditions, further validation is required before extending the conclusions to larger-scale and dynamically changing industrial environments. Full closed-loop online robot localization and control based directly on the fused localization output remain subjects for future investigation.

## 1. Introduction

Autonomous mobile robots and automated guided vehicles (AGVs) require reliable indoor positioning for navigation, docking, fleet coordination, safety monitoring, and digital-twin integration. Particularly in complex industrial or laboratory settings, such as those employing mobile robots for automated sample transport and ambient monitoring, robust and continuous localization is critical [1]. Outdoor satellite navigation cannot be used directly in most industrial buildings because satellite signals are blocked or heavily attenuated by roofs, walls, and machinery. Therefore, indoor positioning systems rely on local infrastructure, including Wi-Fi, Bluetooth, radio-frequency identification, ultra-wideband, infrared, optical, magnetic, and acoustic technologies [2,3,4,5,6,7]. Among these alternatives, ultrasonic positioning is attractive because time-of-flight (ToF) ranging can provide high spatial resolution with inexpensive beacons and direct geometric interpretation [8,9].

The practical limitation of ultrasonic positioning is that sound propagation is strongly shaped by the physical environment. Direct line-of-sight (LoS) paths can be blocked by metal shelves, robot bodies, walls, laboratory furniture, or workers. Reflected paths can be detected later than the direct signal, creating positive range bias and local coordinate divergence. Crossover transitions can also introduce handover jumps, short update-rate drops, and increased jitter when the mobile target moves between acoustic service regions. These effects are visible in the coordinate stream as spikes, discontinuities, and high-frequency jitter or physically impossible jumps. They are especially problematic for AGVs because transient errors near a docking station or narrow corridor can be more damaging than a moderate steady-state bias.

Although ultrasonic positioning systems can achieve high accuracy under favorable conditions, practical deployment in industrial environments remains challenging because acoustic visibility changes dynamically during operation. Existing work has largely focused either on improving ultrasonic ranging accuracy itself or on general-purpose sensor-fusion approaches, while comparatively less attention has been given to lightweight embedded architectures that explicitly address acoustic failure modes such as NLoS outages, crossover handovers, and reflected-path outliers in real robotic deployments. In addition, many localization pipelines rely on external host computers for synchronization, filtering, and logging, which complicates deployment on mobile robotic platforms.

This paper presents an embedded ultrasonic–inertial positioning pipeline for Marvelmind ultrasonic Super-Beacons. The system acquires Marvelmind coordinates and ICM-20948 inertial samples directly on an ESP32-S3-WROOM-1 module, performs real-time packet validation and sliding-window rejection of implausible acoustic samples, and fuses the remaining ultrasonic measurements with high-rate IMU data using a 15-state error-state Kalman filter (ESKF). The embedded design treats ultrasonic localization, inertial prediction, outlier rejection, and trajectory evaluation as one integrated workflow rather than as separate acquisition and post-processing steps. This allows the state estimate to remain continuous through NLoS and crossover tracking gaps while still using valid acoustic positions to bound inertial drift.

The following are the main contributions of this work:

1. A systematic characterization of practical ultrasonic failure modes, including multipath-induced scatter, NLoS acoustic outages, crossover handover jumps, and height-dependent 3D jitter in AGV and robot-arm scenarios;
2. The development of an embedded ultrasonic–inertial localization architecture on an ESP32-S3-WROOM-1 platform, combining packet validation, sliding-window acoustic plausibility gating, and IMU-aided ESKF fusion;
3. A real time-capable implementation using interrupt-driven IMU acquisition, concurrent embedded task separation, and onboard state estimation without dependence on an external processing computer for estimator execution.
4. A repeated-trial evaluation is conducted for both two-dimensional AGV localization and three-dimensional UR10 robot-arm tracking using independent robotic reference trajectories. The NIA and IA configurations are retained as parallel robustness assessments rather than as evidence of the superiority of either acoustic architecture.

The study therefore investigates not only the nominal positioning accuracy of the ultrasonic system but also its robustness during periods of degraded acoustic visibility and intermittent positioning failure. Particular emphasis is placed on trajectory continuity, recovery behavior after acoustic reacquisition, and mitigation of large transient localization errors relevant to industrial robotic operations.

The remainder of this paper is organized as follows. Section 2 reviews related work on ultrasonic indoor positioning, inertial fusion, and embedded localization systems. Section 3 describes the embedded ESP32-S3 architecture, acquisition pipeline, outlier rejection strategy, and ESKF formulation. Section 4 presents the AGV and UR10 experimental scenarios, together with the quantitative localization and runtime evaluation results. Finally, Section 5 and Section 6 discuss the implications and limitations of the proposed approach and summarize the main conclusions.

## 2. Related Work and Technical Background

Indoor localization research commonly distinguishes between fingerprinting-based systems, geometric ranging systems, and multi-sensor fusion systems. Wi-Fi and Bluetooth methods are comparatively inexpensive but usually depend on signal-strength maps, fingerprint databases, or graph-based optimization approaches [4,7,10]. Ultra-wideband systems provide accurate time-based ranging and are widely used in robotics for high-precision real-time tracking but require dedicated anchors, synchronization infrastructure, and careful calibration [5,11]. RFID systems are effective for identification and asset tracking, although their localization precision depends strongly on reader geometry and signal modeling [6].

Comprehensive surveys prove that no single indoor positioning technology dominates all industrial scenarios [3,12,13]. Radio-frequency systems generally scale well and can cover large areas, but their accuracy depends on propagation conditions, antenna geometry, and channel occupancy. Vision and LiDAR can provide rich environmental information, but they increase computational cost and may be affected by illumination, texture, dust, or occlusions. Acoustic systems occupy a complementary design point: they are comparatively inexpensive, geometrically interpretable, and capable of high spatial resolution in calibrated local workspaces. These properties make ultrasonic positioning attractive for laboratories, mobile robot validation, AGV docking studies, and compact industrial work cells where the operating area is known and infrastructure can be installed.

At the same time, practical industrial deployment remains challenging because the quality of acoustic positioning is strongly affected by dynamic environmental conditions. In many real robotic scenarios, localization errors are dominated not by nominal ranging noise but by intermittent failure modes such as reflected-path tracking, acoustic occlusion, sparse update intervals, and abrupt handover behavior between beacon service regions. These effects motivate the need for robustness-oriented localization architectures that combine acoustic positioning with embedded motion-aware estimation.

Table 1 summarizes the main acoustic failure modes considered in this work and links them to the corresponding mitigation stage in the embedded pipeline.

### 2.1. Ultrasonic Indoor Positioning

Ultrasonic systems estimate the distance from acoustic propagation time. In the simplest one-way time-of-flight form commonly used in ultrasonic ranging,

(1) d=cΔt,

where *d* is distance, *c* is the speed of sound, and Δt is the measured time delay [8]. Multiple distances to known beacons allow for trilateration in 2D or 3D. Recent work has improved ultrasonic localization through encoded signals, chirp waveform design, multipath compensation, beacon calibration, synchronization recovery, temperature compensation, robust weak-signal processing, and correction of signal aberrations [14,15,16,17,18,19,20,21]. These contributions demonstrate that ultrasonic systems can achieve centimeter or sub-centimeter performance under controlled conditions.

In commercial ultrasonic systems such as the Marvelmind platform, fixed and mobile beacons exchange ultrasonic pulses and radio-frequency synchronization messages to estimate the mobile position in a local coordinate map. The Super-Beacon can operate either as a stationary or mobile node and supports both non-inverse architecture (NIA) and inverse architecture (IA) modes [22]. In NIA, fixed beacons transmit acoustic signals, and the mobile beacon receives them. In IA, the mobile beacon transmits, and the fixed beacons receive. The two modes offer different trade-offs in terms of the update rate, scalability, and installation complexity. From the perspective of this work, both modes produce a coordinate stream that must be validated before it is used by a robot controller or navigation stack.

The principal limitation of acoustic ToF localization is that sound does not propagate through complex industrial environments as an ideal direct ray. Metallic structures, shelves, vehicle bodies, walls, and nearby machinery can create reflected propagation paths. If the direct path is blocked or attenuated, the receiver may lock onto a reflected path or lose the signal entirely. Because reflected paths are longer than the direct path, they tend to create positive range bias that appears as coordinate jumps, local trajectory bending, delayed recovery, or short periods of complete localization failure. These physical effects are particularly problematic for AGVs and mobile robots because transient localization discontinuities can be more critical than moderate steady-state positioning errors. This motivates the explicit failure-mode analysis and mitigation strategy investigated in the present work.

To address these environmental challenges, recent advancements increasingly incorporate deep learning and adaptive filtering into the localization pipeline. For example, graph neural network-based multimodal fusion models have been proposed to mitigate non-line-of-sight (NLOS) conditions by learning complex environmental propagation characteristics directly from measurement data [23]. These modern approaches emphasize moving beyond static channel assumptions, instead dynamically adjusting the weight of acoustic measurements based on real-time signal reliability and multipath interference.

### 2.2. Inertial Fusion and Error-State Filtering

The remaining challenge is robustness under dynamically changing acoustic visibility. Hardware-level improvements alone cannot always remove transient acoustic failures, especially when the direct path is fully blocked. Therefore, sensor fusion is a natural extension in terms of maintaining trajectory continuity during periods of degraded acoustic conditions. Inertial measurement units (IMUs) provide high-rate acceleration and angular-rate measurements that are locally continuous, independent of acoustic visibility, and already available in many mobile sensing platforms. Their principal weakness is integration drift over time. Ultrasonic measurements, in contrast, provide bounded absolute position information when available but may become sparse, delayed, or corrupted during NLoS intervals. Therefore, these sensing modalities are complementary: inertial data preserve short-term motion continuity, while ultrasonic measurements constrain long-term drift. An error-state Kalman filter (ESKF) is well suited to this structure because it propagates position, velocity, attitude, and sensor biases with inertial measurements while correcting accumulated drift whenever reliable absolute position updates become available.

Error-state filtering is widely used in inertial navigation because it separates the nominal state from a small perturbation state. This is particularly advantageous for attitude estimation, where the physical orientation is represented by a unit quaternion while the covariance can be expressed with a minimal three-dimensional attitude-error representation. Sola’s quaternion kinematics formulation provides a widely used derivation of the quaternion-based ESKF propagation and correction [24]. Similar principles are used in visual–inertial navigation and robotic state-estimation systems that combine high-rate inertial propagation with lower-rate absolute or relative measurements [25]. The present work applies this concept specifically to ultrasonic indoor positioning under intermittent acoustic visibility. In contrast to purely smoothing-based approaches, the embedded ESKF maintains a dynamic motion estimate during acoustic outages and reintroduces ultrasonic corrections only after plausibility validation. This makes the approach particularly suitable for AGV stop–start motion, docking maneuvers, and robotic trajectories with temporary NLoS conditions or sparse acoustic updates.

Recent navigation research emphasizes the critical distinction between loosely coupled and tightly coupled architectures. While loosely coupled systems fuse independently processed position coordinates with IMU data, tightly coupled systems fuse raw range measurements directly, which improves robustness when the number of visible beacons drops below the threshold required for standalone positioning. Furthermore, modern multi-sensor fusion architectures increasingly rely on factor graph optimization (FGO) to handle asynchronous measurements and nonlinear drift over long intervals. However, FGO can be computationally demanding for lightweight embedded platforms. Alternatively, adaptive and tightly coupled architectures increasingly incorporate ML-based NLOS detection or gradient-adaptive techniques to provide similar resilience against NLOS conditions without the full computational overhead of a sliding-window graph [26,27]. The present work adopts the ESKF approach, prioritizing low-latency real-time embedded execution on microcontrollers while relying on dedicated kinematic gating to achieve comparable outlier resilience.

### 2.3. Embedded Localization Nodes

For AGVs or mobile robots, the localization pipeline should not depend on a high-power external computer for basic state propagation and measurement validation. Embedded processing reduces communication latency, simplifies deployment, and allows the sensing node to provide a compact fused localization stream directly to a robot controller. It also improves robustness against unstable network conditions or temporary diagnostic-computer disconnection during operation. The ESP32-S3-WROOM-1 module is a low-cost Wi-Fi/Bluetooth microcontroller platform with sufficient peripheral support for UART, I2C, and network communication. The principal technical specifications of the selected ESP32-S3-WROOM-1 module are detailed in Table 2 [28]. ESP-IDF provides the official software-development framework and hardware abstraction layer for ESP32-S3 applications [29]. The ICM-20948 integrates a three-axis gyroscope, three-axis accelerometer, and three-axis magnetometer in a compact, nine-axis motion-tracking device [30]. Together, these components provide a practical basis for implementing a lightweight embedded ultrasonic–inertial localization node with onboard synchronization, packet validation, outlier rejection, and state estimation. Compared with host-computer-based localization pipelines, such an embedded architecture allows time-critical prediction and correction steps to remain directly coupled to the sensor interfaces. This is particularly relevant for intermittent ultrasonic positioning, where delayed processing or irregular synchronization can further amplify transient localization failures. Therefore, the architecture investigated in this work treats embedded acquisition, plausibility filtering, inertial propagation, and ultrasonic correction as one integrated localization pipeline rather than as separate post-processing stages.

## 3. Materials and Methods

### 3.1. System Architecture

The experimental platform consists of Marvelmind ultrasonic Super-Beacons, a mobile sensing unit, an ESP32-S3-WROOM-1 embedded controller, and a browser-based visualization and logging interface. The ultrasonic system can operate in non-inverse architecture (NIA) mode, where stationary beacons transmit and the mobile beacon receives, or inverse architecture (IA) mode, where the mobile beacon transmits and stationary beacons receive. Both architectures output coordinate estimates in a local Marvelmind reference frame. For all experimental scenarios evaluated in this study, the position was determined using time-of-flight (ToF) multilateration based on distance measurements from a local constellation of four stationary ultrasonic beacons rather than relying on signal-strength path-loss models. Regarding electromagnetic compatibility (EMC) and spectrum occupancy, the Super-Beacons exchange synchronization messages using the 915 MHz radio-frequency band. In contrast, the remaining wireless devices in the experimental environment, including the ESP32-S3 diagnostic interface and external laboratory equipment, operate on standard 2.4 GHz and 5 GHz Wi-Fi networks. This frequency separation ensures that no radio-frequency interference or bandwidth contention occurs between the ultrasonic positioning infrastructure and the surrounding wireless systems.

To provide a comprehensive overview of the acoustic infrastructure, Table 3 summarizes the core hardware specifications, operating range, and signal conditions of the Marvelmind Super-Beacon used in this study [22].

The embedded localization node was designed to perform time-critical acquisition, validation, and state-estimation tasks directly on the mobile sensing platform rather than on an external host computer. This architecture reduces communication dependency during operation and allows localization continuity to be maintained even when diagnostic services or external logging interfaces are temporarily unavailable.

**Figure 1:**
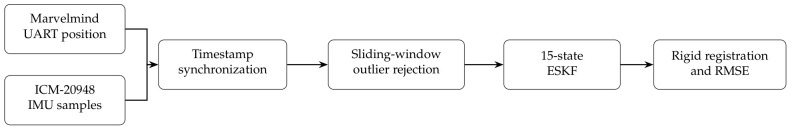
Embedded processing pipeline for ultrasonic positioning with integrated IMU-aided ESKF correction on ESP32-S3.

The experimental firmware is implemented in Rust with ESP-IDF. Marvelmind position packets are acquired through UART2 at 500 kbaud, validated with a MODBUS CRC16 integrity check, and converted from millimeters to meters before entering the estimator pipeline. The ICM-20948 IMU is connected through I2C0 at 400 kHz and sampled from a hardware data-ready interrupt at 500 Hz. Before online operation, a static initialization phase estimates gyroscope bias, acceleration scaling, and the gravity-alignment quaternion required for inertial propagation. In addition to the estimator tasks, the ESP32-S3 hosts a local Wi-Fi access point, an embedded HTTP server, and a WebSocket-based diagnostic stream for monitoring and logging. The overall processing sequence is summarized in Figure 1. Raw ultrasonic coordinates and IMU measurements are synchronized locally on the embedded controller, implausible acoustic outliers are rejected by a kinematic sliding-window gate, accepted coordinates are fused with inertial data in an ESKF, and the resulting trajectory is compared with independent robotic references during offline evaluation.

**Figure 2:**
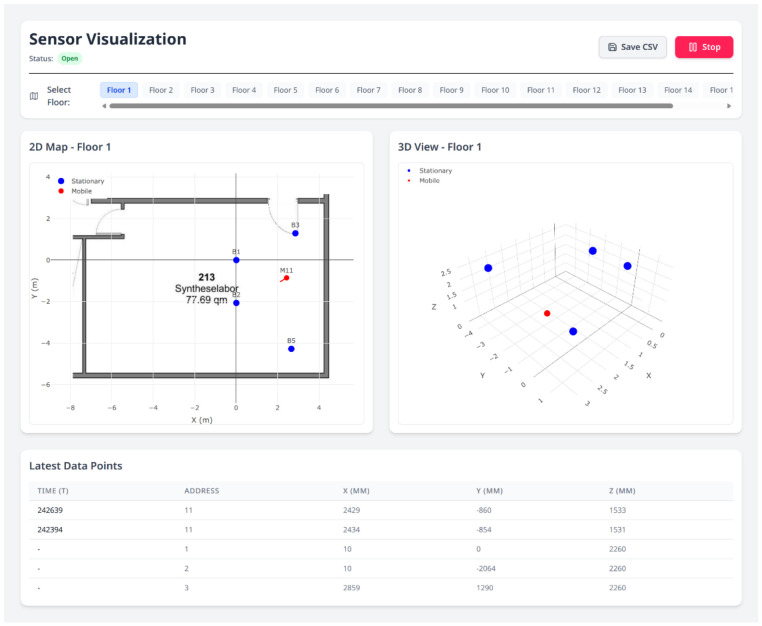
Example of the browser-based diagnostic WebUI used for live ultrasonic position monitoring and CSV data export. The interface visualizes stationary and mobile beacons in 2D and 3D and lists the latest received coordinate packets from the embedded ESP32-S3 stream.

Figure 2 shows the used browser-based diagnostic interface, together with the embedded WebSocket stream. The interface visualizes the selected floor map, live 2D and 3D beacon positions, the current mobile node, and the most recent coordinate packets, in addition to providing CSV export functionality for experimental logging. The WebUI was used exclusively for monitoring and data recording during experiments; the localization estimator, itself, executed continuously on the embedded controller. Quantitative evaluation and trajectory registration against the robotic ground truth were subsequently performed offline to ensure repeatable comparison across trials.

**Figure 3:**
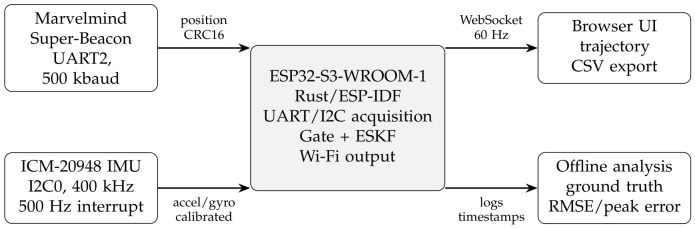
Functional hardware topology of the embedded ultrasonic–inertial localization node. The ESP32-S3 acquires ultrasonic and inertial measurements locally and publishes diagnostic output to the browser WebUI, while ground-truth registration and RMSE analysis are performed offline.

Figure 3 summarizes the embedded hardware topology. The ESP32-S3 acts as the central acquisition and estimation node. It receives validated ultrasonic position packets through UART2, samples the ICM-20948 IMU through I2C0, performs onboard prediction and correction, and publishes the fused localization state through the local Wi-Fi interface. The figure is intentionally drawn as a functional architecture diagram to emphasize the separation between embedded estimation, communication services, and offline evaluation.

Table 4 summarizes the embedded implementation used in the experiments. Table 5 summarizes the hardware and mounting parameters used in the experiments. Electrical interfaces, communication rates, and sampling frequencies follow the embedded ESP32-S3 firmware configuration, while the mounting rows document how the same sensing node was installed on the AGV carrier and the UR10 wrist-joint fixture. These parameters are required to reproduce the lever-arm compensation and for interpretation of ultrasonic measurements relative to the IMU body frame.

**Figure 4:**
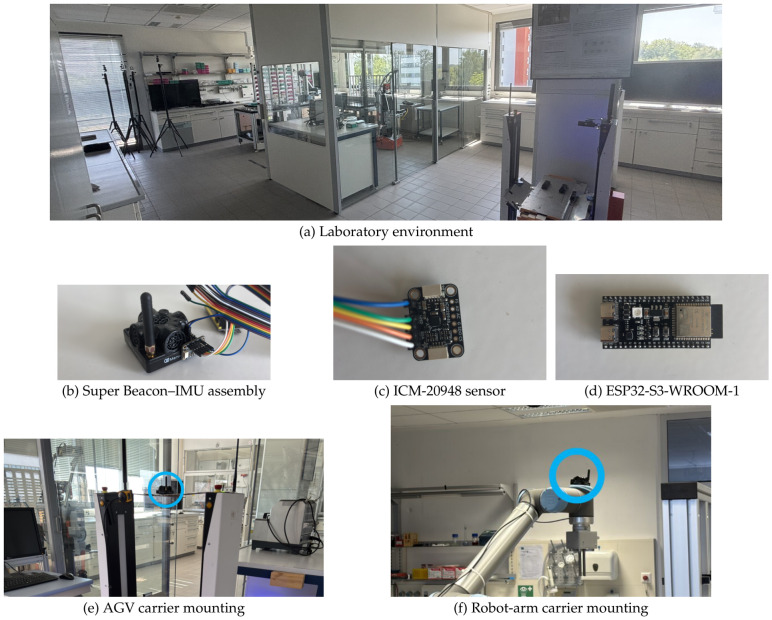
Photographic documentation of the experimental environment, embedded sensing hardware, and carrier mounting configurations. The ultrasonic–inertial sensing node was mounted on the AGV platform for 2D mobile tests and above the UR10 wrist joint for controlled 3D trajectory evaluation; the wrist joint was kept fixed so that the sensor-to-end-effector transform remained constant. The blue circles in (**e**,**f**) indicate the mounting positions of the sensors on the AGV and the robotic arm, respectively.

Figure 4 documents the corresponding physical setup. Panel (a) shows the laboratory environment used for the acoustic positioning experiments, panels (b)–(d) show the sensing hardware, and panels (e) and (f) show the two carrier configurations referenced in Table 5. In the UR10 tests, the sensor node was mounted above the wrist joint rather than directly on the end-effector to preserve the line of sight (LoS) to the ultrasonic beacon field. During all robot-arm trials, the wrist joint remained fixed so that the rigid transform between the sensing node and the end-effector remained constant throughout the trajectory.

**Figure 5:**
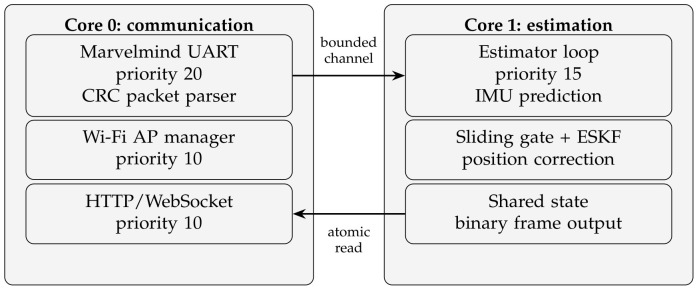
Firmware task organization on the ESP32-S3. Core 0 handles communication services, including the Marvelmind UART parser, Wi-Fi access-point manager, and HTTP/WebSocket server. Core 1 runs the estimator path, where IMU prediction is followed by sliding-window acoustic gating, ESKF position correction, and shared-state publication. The bounded channel transfers validated Marvelmind packets from the UART task to the estimator, while the WebSocket server reads the published state atomically for diagnostic visualization and CSV logging.

Figure 5 shows the concurrent firmware task organization implemented on the ESP32-S3. The firmware explicitly separates communication-oriented tasks from estimator-oriented tasks. Core 0 handles the Marvelmind UART parser, Wi-Fi access-point management, and HTTP/WebSocket services, while Core 1 prioritizes IMU-driven prediction, acoustic measurement gating, ESKF correction, and publication of the latest fused state. This separation prevents Web-serving or Wi-Fi activity from interfering with the high-rate IMU processing path.

The task priorities in Figure 5 reflect the timing requirements of the embedded localization node. The Marvelmind UART parser receives the highest communication priority because incomplete or delayed serial parsing can corrupt packet framing. The estimator task is isolated on the second core so that 500 Hz IMU propagation remains independent of Wi-Fi traffic, browser requests, and diagnostic streaming. The bounded communication channel between the UART parser and estimator prevents unbounded memory growth during burst-like packet arrival, while the atomic shared-state access allows the WebSocket service to publish diagnostic data without blocking the estimator loop.

### 3.2. Embedded Acquisition and Synchronization

The embedded implementation was designed to move the time-critical components of the positioning pipeline from the host computer directly onto the mobile sensing node. This architectural decision is particularly important for AGV and robotic applications because localization must remain available even when a diagnostic computer is disconnected, network traffic is unstable, or acoustic updates arrive irregularly due to environmental occlusion or beacon handover effects. The ESP32-S3-WROOM-1 provides sufficient computational and peripheral resources for this purpose: one processing core can handle communication-oriented services, while the second core prioritizes IMU acquisition and estimator execution.

The Marvelmind interface is implemented as a dedicated UART acquisition task. Incoming bytes are accumulated until a complete position frame is available. A frame is accepted only when the expected packet structure is detected and the MODBUS CRC16 integrity check is valid. The embedded parser extracts the timestamp and Cartesian coordinates, converts coordinates from millimeters to meters, and forwards the resulting measurement to the estimator through a bounded communication channel. This packet-level validation acts as the first protection layer in the localization pipeline: malformed or corrupted serial frames are discarded before they can influence either the sliding-window plausibility or the ESKF state estimate.

The IMU acquisition path is interrupt-driven. The ICM-20948 is configured for a 500 Hz sampling rate, and each hardware data-ready event triggers acquisition of accelerometer and gyroscope measurements. Before online operation, the sensing unit undergoes a short static calibration phase. During this phase, the firmware averages 200 stationary samples to estimate the gyroscope bias, computes acceleration scaling from the local gravity magnitude, and determines a gravity-alignment quaternion used for inertial initialization. The resulting calibrated IMU sample contains acceleration and angular-rate measurements in a frame suitable for inertial propagation. This calibration step is essential because even small gyroscope bias or gravity misalignment errors can accumulate rapidly during acoustic outages. Therefore, in intermittent ultrasonic positioning scenarios, incorrect inertial initialization may appear as false motion during prediction-only intervals and significantly degrade recovery behavior after acoustic reacquisition.

The estimator combines two asynchronous streams with different temporal characteristics. IMU measurements arrive at a high and nearly regular rate, while ultrasonic position updates arrive more slowly and may become sparse or temporarily unavailable during NLoS or crossover transitions. Therefore, the estimator treats IMU measurements as the prediction clock and treats ultrasonic coordinates as opportunistic absolute corrections. When no validated acoustic measurement is available, inertial prediction continues uninterrupted. When a new ultrasonic coordinate arrives, it is first processed by the kinematic sliding-window gate and is used for ESKF correction only if the measurement is considered physically plausible. This architecture reflects the complementary behavior of the sensors themselves: inertial measurements provide continuous short-term motion information but accumulate drift over time, whereas ultrasonic coordinates provide bounded absolute position information but may become intermittent or corrupted under unfavorable acoustic conditions. Therefore, the embedded fusion pipeline is designed to preserve trajectory continuity during temporary acoustic degradation while still constraining long-term drift whenever valid ultrasonic measurements return.

For monitoring and experimental logging, the firmware exposes a compact fused-state stream through a WebSocket endpoint. Each binary message contains timestamped position components, together with the most recent estimator state. A browser-based diagnostic interface can display the live trajectory, visualize beacon geometry, and export recorded measurements for offline evaluation. In the experiments reported here, the embedded localization stream and the independent robotic references were aligned offline to compute RMSE, peak failure-interval error, and recovery behavior after acoustic reacquisition. The offline evaluation stage does not alter the real-time estimator execution itself. Instead, it provides a repeatable comparison against independent robotic references. This separation between embedded runtime estimation and offline quantitative analysis allows the localization architecture to be evaluated reproducibly without introducing host-side corrections into the real-time processing path. When recorded datasets are reprocessed for comparison, the firmware-consistent replay uses the identical gating, inertial propagation, ultrasonic correction, and state-publication logic implemented in the ESP32-S3 firmware; only the quantitative comparison against robotic references is performed offline.

### 3.3. Ground-Truth Platforms

Two independent robotic references were used for quantitative evaluation. The references were employed exclusively for offline trajectory comparison and were not used by the embedded estimator during runtime operation. They serve as repeatable trajectory references for comparative robustness evaluation rather than as metrological-grade absolute motion-capture measurements.

In 2D experiments, a mobile AGV carried the mobile Ultrasonic–inertial sensing node above the robot chassis. The AGV followed predefined trajectories through multiple acoustic service regions and parking locations representative of typical indoor navigation and docking scenarios. A network-based polling interface recorded the robot’s reference position, together with timestamps, and provided the planar ground-truth trajectory used for evaluation.

In the 3D experiments, a UR10 robot arm carried the mobile sensing node above its wrist joint rather than directly on the end-effector. This mounting configuration was selected to maintain LoS visibility between the ultrasonic sensor and the stationary beacon field during the programmed trajectories. The wrist-joint orientation remained fixed during each path execution such that the rigid transform between the sensing node and the end-effector remained constant and could be modeled as a static offset during evaluation. The UR10 executed predefined Cartesian trajectories between known spatial nodes with controlled motion speed. The corresponding reference trajectory was reconstructed by timestamped interpolation between commanded node positions. This approach provided a repeatable and geometrically well-defined reference path for evaluating spatial-tracking continuity, vertical-axis behavior, and recovery after acoustic degradation without requiring an external motion-capture system. The purpose of the UR10 reference was not to establish metrological absolute positioning accuracy at motion-capture precision but, rather, to provide a stable and repeatable robotic reference for comparative evaluation of raw ultrasonic positioning, sliding-window filtering, and embedded IMU-ESKF mitigation under identical trajectory conditions. Because all evaluated localization variants were compared against the same robotic reference, synchronization pipeline, and registration procedure, the setup allows for consistent analysis of relative error reduction and recovery behavior across repeated trials.

Both references were treated as external evaluation systems rather than as components of the localization algorithm itself. The AGV reference provides a planar trajectory, together with robot-controller timestamps, while the UR10 reference provides a repeatable programmed Cartesian path with known node positions and timing constraints. The embedded estimator receives only IMU measurements and ultrasonic coordinates; the robotic references are introduced exclusively during offline evaluation after estimator execution has completed.

To compare the embedded localization output with the robotic references, the ultrasonic–inertial trajectory was temporally aligned and rigidly registered only during post-processing. This separation between embedded estimation and offline reference comparison is important because it prevents the reference systems from influencing the real-time filter state or artificially improving localization performance during runtime.

**Figure 6:**
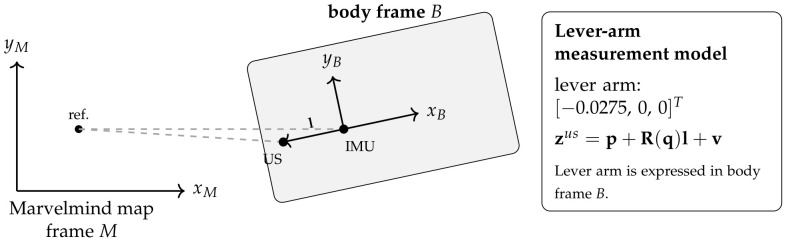
Coordinate-frame relationship for the calibrated lever-arm configuration. The ultrasonic acoustic reference point is offset from the IMU origin by $l={\left[-0.0275,\;0,\;0\right]}^{T}$ in body frame *B*; the schematic offset is enlarged for readability.

Figure 6 summarizes the coordinate-frame relationship used for the calibrated lever-arm configuration. The ultrasonic acoustic reference point is offset from the IMU origin by a fixed body-frame lever arm, which is included explicitly in the ESKF measurement model.

### 3.4. Error Characterization

The raw ultrasonic coordinate stream was analyzed with respect to three dominant failure modes observed during the experiments. Multipath scattering was identified when the coordinate stream exhibited local deviations, increased scatter, or delayed recovery while the physical carrier followed a smooth trajectory. NLoS occlusion was identified when direct acoustic paths were blocked by obstacles and the localization output showed isolated spikes, sparse updates, or longer trajectory divergence. Crossover-zone signal degradation was identified during transitions between acoustic service regions, where beacon visibility and localization geometry changed abruptly and produced temporary handover artifacts.

For each trial, the ultrasonic or fused localization samples were temporally matched to the corresponding robotic reference trajectory. Because the ultrasonic coordinate frame and the robot reference frame are not inherently identical, trajectories were rigidly aligned before error calculation. Let $p_{i}$ denote an ultrasonic or fused localization point and $g_{i}$ denote the corresponding ground-truth point. Following the closed-form least-squares point-pattern alignment approach proposed by Umeyama [31], a rigid transform ((R,t)) was estimated using singular value decomposition (SVD) to minimize the spatial registration error between the two point sets.

(2) $$\sum_{i}{∥Rp_{i}+t-g_{i}∥}_{2}^{2}.$$

The pointwise localization error and root mean square error (RMSE) were then computed after this rigid registration as follows:

(3) $$E_{i}={∥Rp_{i}+t-g_{i}∥}_{2},\;\mathrm{RMSE}=\sqrt{\frac{1}{N}\sum_{i=1}^{N}E_{i}^{2}}.$$

This evaluation procedure removes constant coordinate-frame offsets and mounting differences so that the reported error reflects localization quality rather than arbitrary global frame placement. The registration step is especially important for comparing repeated AGV and UR10 trials because the embedded sensing node and the robotic reference systems do not share a common physical origin.

No external calibrated tracking system was available during the experiments. Consequently, the AGV and UR10 trajectories were treated as repeatable robotic reference trajectories rather than metrological-grade absolute ground truth. The associated reference uncertainty was estimated based on the manufacturer specifications of the respective robotic platforms. According to the UR10 datasheet, the robot exhibits a repeatability of ±0.1 mm [32], whereas the ProBOT L mobile platform specifies a positioning accuracy of approximately ±5 mm and $3^{\circ }$ under nominal operating conditions [33]. These uncertainties are substantially smaller than the raw ultrasonic positioning errors and the replay-based fused localization errors reported in this study. While the reference uncertainty may contribute slightly to the absolute RMSE values, particularly for the 2D AGV experiments, it is unlikely to affect the relative performance comparison between raw ultrasonic localization, sliding-window filtering, LKF smoothing, and firmware-consistent IMU-ESKF replay.

In addition to trial-mean RMSE, particular attention was given to transient failure behavior. Short-duration localization discontinuities can be significantly more critical for AGV docking, obstacle avoidance, and robotic trajectory execution than moderate steady-state bias. Therefore, the analysis emphasizes peak-error intervals, recovery behavior after acoustic reacquisition, and the persistence of large deviations during NLoS or crossover transitions. These metrics are intended to characterize not only nominal positioning accuracy but also the robustness and continuity of the embedded localization architecture under degraded acoustic conditions.

### 3.5. Sliding-Window Outlier Rejection

The first mitigation step rejects implausible ultrasonic outliers before they are introduced into the ESKF correction step. The purpose of this stage is not to improve nominal low-noise localization accuracy but to prevent severe reflected-path measurements, handover artifacts, or transient NLoS coordinates from destabilizing the probabilistic estimator. Let $z_{k}^{us}={\left[x_{k},y_{k},z_{k}\right]}^{T}$ denote the ultrasonic position measurement at time step *k*. The instantaneous displacement relative to the previously accepted ultrasonic coordinate provides a velocity estimate:

(4) $$v_{k}=\frac{∥z_{k}^{us}-z_{k-1}^{us}{∥}_{2}}{\Delta t_{k}}.$$

If $v_{k}$ exceeds a physically feasible threshold for the carrier platform, the measurement is rejected. A second acceleration-based plausibility constraint removes measurements that do not violate the velocity threshold directly but imply unrealistic changes in motion between consecutive updates. The remaining accepted measurements are processed by a sliding statistical window of the last N=10 valid samples. For each coordinate axis, the local mean and standard deviation are estimated according to the accepted measurements only. A measurement is rejected whenever its Z-score exceeds the threshold of $z_{\max}=3.0$ in any coordinate component, following the general normalized-residual principle commonly used in classical outlier testing [34]. This layered rejection strategy prevents severe acoustic outliers from biasing the ESKF state estimate or artificially inflating the filter covariance. The gate is designed specifically for intermittent acoustic localization, where reflected-path measurements may still appear geometrically plausible in isolation but imply physically impossible motion relative to the carrier dynamics.

The rejection thresholds are selected from the carrier dynamics rather than from nominal ultrasonic sensor specifications alone. For the AGV experiments, the admissible velocity and acceleration limits are derived from commanded robot motion, together with a conservative safety margin. For the UR10 robot-arm experiments, the limits are determined according to the programmed trajectory, known interpolation timing, and expected sampling interval. This approach prevents the estimator from accepting reflected-path coordinates simply because they remain numerically close to previous samples while still violating the physically achievable motion of the carrier. The gate is intentionally conservative. In the context of industrial localization, rejecting an occasional valid acoustic update is generally less harmful than introducing a large NLoS outlier into the estimator state. A single reflected-path coordinate can otherwise pull the nominal state away from the physical trajectory and increase recovery time after acoustic reacquisition.

The sliding-window formulation also stabilizes the local statistical behavior of the gate. A single global rejection threshold would be too rigid because the acoustic coordinate variance changes with beacon geometry, carrier orientation, and environmental visibility conditions. Near well-conditioned beacon constellations, the coordinate stream exhibits low variance, and even small reflected-path deviations should be rejected. Near crossover boundaries or partially degraded acoustic geometry, temporary variance increases are expected and should not automatically invalidate the trajectory. By updating the local mean and standard deviation according to recently accepted measurements only, the gate adapts to local noise conditions while preventing rejected outliers from contaminating the statistical window itself. Therefore, the resulting behavior is locally adaptive rather than globally fixed.

Within the embedded localization pipeline, rejected ultrasonic measurements are excluded from the ESKF-correction step, but estimator propagation continues uninterrupted. During rejected or missing acoustic intervals, the ESKF prediction step continues at the IMU rate using inertial propagation alone. The next accepted ultrasonic measurement can then correct the accumulated inertial drift without introducing a discontinuity into the trajectory estimate. This behavior is particularly important in industrial environments where acoustic visibility may change abruptly because of shelves, machinery, robot structures, personnel movement, or temporary occlusion. Therefore, the sliding-window gate acts as a lightweight measurement-quality classifier positioned before the probabilistic fusion stage, separating communication integrity, physical plausibility, and estimator correction into distinct processing layers.

### 3.6. Embedded IMU-Aided Error-State Kalman Filter

The embedded fusion module uses an error-state Kalman filter (ESKF) rather than a simpler constant-acceleration extended Kalman filter (EKF). The selected formulation follows established quaternion-based inertial-navigation and error-state filtering approaches [24,35] while remaining computationally compatible with the ESP32-S3 embedded target. The ESKF was selected because it allows for continuous inertial propagation during temporary acoustic outages while maintaining a compact and numerically stable state representation suitable for real-time embedded execution. The nominal filter state is defined as

(5) $$x=\{p,v,q,b_{a},b_{g}\},$$

where p is position; v is velocity; q is the body-to-world unit quaternion; and $b_{a}$ and $b_{g}$ denote accelerometer and gyroscope biases, respectively. The filter covariance is maintained over the corresponding 15-dimensional error state:

(6) $$\delta x={\left[\delta p,\delta v,\delta \theta ,\delta b_{a},\delta b_{g}\right]}^{T}.$$

After static IMU calibration, the prediction step removes the estimated sensor biases from the measured acceleration ($a_{m}$) and angular rate ($\omega_{m}$). With the gravity vector ($g={\left[0,0,-9.80665\right]}^{T}$), the world-frame acceleration becomes

(7) $$a_{w}=R\left(q\right)\left(a_{m}-b_{a}\right)+g.$$

The nominal state is propagated using standard strapdown inertial kinematics on the quaternion manifold [24,35]:

(8) $$\begin{array}{cc} p_{k+1} & =p_{k}+v_{k}\Delta t+\frac{1}{2}a_{w}\Delta t^{2},\\ v_{k+1} & =v_{k}+a_{w}\Delta t,\\ q_{k+1} & =q_{k}⊗\mathrm{Exp}\left(\left(\omega_{m}-b_{g}\right)\Delta t\right). \end{array}$$

The covariance prediction uses the discretized error-state transition matrix. Its dominant blocks model position-velocity coupling, attitude sensitivity to specific force, accelerometer-bias coupling, gyroscope-driven attitude propagation, and gyroscope-bias evolution. Process noise is constructed from accelerometer white noise, gyroscope white noise, accelerometer-bias random walk, and gyroscope-bias random walk parameters. To improve numerical robustness on the embedded target, the covariance matrix is explicitly symmetrized after prediction.

When a valid ultrasonic position becomes available, the measurement model includes the rigid lever arm (l) between the IMU frame and the ultrasonic acoustic reference point. The corresponding coordinate relationship is summarized in Figure 6. The ultrasonic measurement model follows the standard rigid-body relation between the inertial body frame and an offset measurement point [24,35]:

(9) $$z_{k}^{us}=p_{k}+R\left(q_{k}\right)l+v_{k}.$$

Therefore, the linearized measurement matrix contains an identity block for position, together with an attitude-dependent block proportional to $-R\left(q\right){\left[l\right]}_{\times }$. The correction stage follows the standard Kalman filtering formulation [36]. The Kalman gain is computed as

(10) $$K_{k}=P_{k|k-1}H_{k}^{T}{\left(H_{k}P_{k|k-1}H_{k}^{T}+R_{k}\right)}^{-1},$$

and the covariance is updated using the Joseph stabilized form to improve numerical conditioning in single-precision arithmetic. The resulting error vector is injected into the nominal state: position and velocity are updated additively, attitude is corrected through quaternion multiplication, and accelerometer and gyroscope biases are corrected additively. The error state is subsequently reset before the next prediction interval.

If the ultrasonic sample is rejected by the plausibility gate or temporarily unavailable because of acoustic dropout, the measurement update is skipped, and only inertial propagation is retained:

(11) $$x_{k|k}=x_{k|k-1},\;P_{k|k}=P_{k|k-1}.$$

This behavior represents a key difference relative to the earlier sliding-window linear Kalman baseline. Acoustic outages are not bridged solely through constant-velocity extrapolation but through inertial motion propagation intrinsic to the sensing unit and executed directly on the ESP32-S3 estimator.

The error-state formulation was selected specifically for embedded implementation because the attitude perturbation remains locally small, even when the nominal quaternion represents larger physical rotations. This avoids direct filtering of the four quaternion components as independent, unconstrained states and simplifies quaternion normalization after correction. Therefore, the nominal state carries the physically meaningful trajectory estimate, while the covariance describes only small perturbations around this nominal motion.

Single-precision floating-point arithmetic is used throughout the estimator to reduce computational cost and maintain the real-time capability of the ESP32-S3 platform. This choice makes numerical conditioning particularly important. Therefore, the implementation applies covariance symmetrization after prediction and Joseph-form covariance correction after measurement updates to reduce the accumulation of numerical asymmetry over long runtime intervals. The initial covariance configuration assigns greater uncertainty to position and velocity states than to attitude and bias states. This reflects the initialization process: the first valid ultrasonic measurement establishes the initial absolute position estimate, whereas static IMU calibration already provides approximate initial orientation and sensor-bias estimates. The ultrasonic measurement covariance is parameterized according to the expected acoustic positioning uncertainty. In the current implementation and evaluation, this parameter is fixed at a constant empirical value (as listed in Table 6) to evaluate the baseline ESKF performance without introducing confounding variables.

The lever-arm compensation term is included because the IMU origin and the ultrasonic acoustic reference point are not necessarily co-located physically on the sensing node. During rotational motion, a nonzero lever arm changes the expected acoustic measurement position, even when the IMU pose estimate, itself, remains correct. Therefore, ignoring this offset would introduce systematic geometric errors during AGV turning maneuvers or robot-arm motion. Including the lever arm in the measurement model allows the same estimator architecture to be used consistently for both AGV-mounted and robot-arm-mounted sensing configurations.

Table 6 summarizes the ESKF and sliding-window gating parameters used during the embedded evaluation. The IMU noise values in Table 6 correspond to the ESP32-S3 ESKF firmware configuration used during the experiments. The standard deviation of ultrasonic measurement is initialized to 3 cm, matching the expected order of magnitude of the positioning accuracy of Marvelmind Super-Beacons under favorable local geometry. The velocity and acceleration rejection thresholds are intentionally conservative for the relatively low-speed AGV and UR10 trajectories evaluated in this study. Their primary purpose is to reject physically implausible reflected-path measurements while preserving normal acceleration, deceleration, and turning behavior. The final parameter set was kept fixed for all NIA and IA runs; no scenario-specific retuning was applied to the reported datasets. The lever arm is configured as $l={\left[-0.0275,\;0,\;0\right]}^{T}$ m, representing the measured body-frame offset between the IMU origin and the ultrasonic acoustic reference point in the current sensing-node configuration. A physically meaningful removal of the IMU from the proposed 15-state ESKF would fundamentally alter the estimator structure. The ESKF propagation model is driven by measured acceleration and angular rates; without these inputs, the velocity, attitude, and bias states lose their physical interpretation, and the estimator effectively reduces to a kinematic position filter with constant-velocity prediction. For this reason, the sliding window-plus-LKF configuration is used as the non-inertial control case throughout the evaluation.

## 4. Experiments and Results

### 4.1. Experimental Scenarios

Four experimental categories were used: 2D AGV tracking under a non-inverse architecture (NIA), 2D AGV tracking under an inverse architecture (IA), 3D robot-arm tracking under an NIA, and 3D robot-arm tracking under an IA. The same embedded sliding-window and IMU-ESKF pipeline was applied consistently to all scenarios, with particular attention given to NLoS intervals, crossover transitions, and recovery behavior after degraded acoustic visibility. Table 7 summarizes the overall evaluation design and corresponding reference systems.

**Figure 7:**
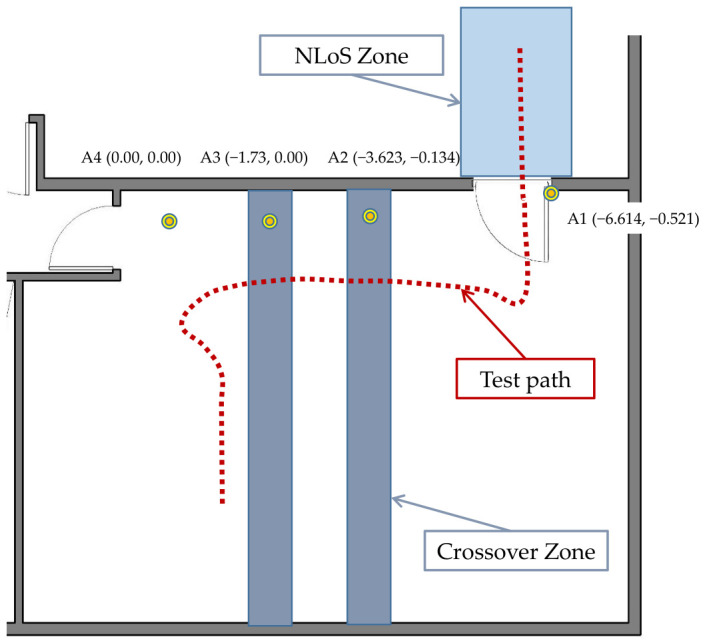
Experimental 2D AGV layout used for ultrasonic indoor positioning evaluation, matching the planned layout in the experimental presentation. The dark-red dashed curve denotes the AGV ground-truth route from the lower-left area toward the upper-right area. The gray regions mark crossover zones between acoustic service areas, while the blue region marks the NLoS zone.

The 2D AGV experiments represent the scenario most relevant to practical industrial mobile robots. The ESP32-S3 ultrasonic–inertial sensing node was mounted directly on the AGV platform, and the vehicle followed predefined trajectories through a beacon-covered laboratory environment. The physical dimensions of the laboratory were 11.454 m by 7.26 m, providing a total indoor test area of approximately 83.16 m^2^. To maintain a strictly controlled acoustic environment and prevent unmodeled dynamic occlusions, no human operators or personnel were present in the workspace during the measurements; both the AGV and the UR10 robotic arm executed their motions autonomously according to pre-programmed control sequences. The trajectory intentionally traversed multiple acoustic conditions, including stable LoS regions, transitions between adjacent acoustic service zones, and an adjacent room/area outside the ultrasonic sensor coverage map used to emulate NLoS acoustic outage. This setup allowed for evaluation not only of nominal positioning accuracy but also of localization continuity and failure behavior during periods of degraded acoustic visibility. Figure 7 shows the experimental 2D AGV layout. The trajectory was selected specifically to expose the localization system to acoustic handover effects and intermittent visibility changes representative of realistic indoor AGV operation.

The crossover and NLoS intervals were defined a priori based on the experimental ultrasonic coverage map and laboratory layout prior to any localization-error analysis. The crossover zones correspond to the predesigned transition regions between adjacent ultrasonic service areas within the configured Marvelmind map, where measurements from neighboring beacon groups may overlap or change dominance. The NLoS region was intentionally defined as an adjacent area outside the ultrasonic sensor coverage map. When the AGV entered this region, ultrasonic measurements were expected to become unavailable, intermittent, or significantly degraded, thereby creating a controlled acoustic-outage scenario representative of non-line-of-sight operation. For the subsequent analysis, zone labels were assigned by intersecting the time-stamped robotic reference trajectory with these predefined spatial regions. Importantly, the crossover and NLoS intervals were not determined retrospectively according to the observed localization errors, estimated trajectories, or performance metrics. Furthermore, the assigned region labels were not used by the localization algorithm during filtering, state estimation, or firmware-consistent replay. Their sole purpose was to support failure-mode analysis, visualization, region-specific performance assessment, and peak-error evaluation. 

**Figure 8:**
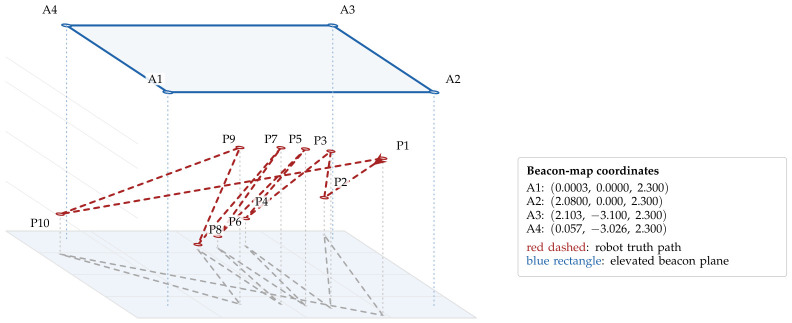
Experimental 3D UR10 robot-arm plan and programmed truth path. The red dashed polyline shows the closed Cartesian reference trajectory through P1–P10 and back to P1, while the gray dashed curve shows its projection on the horizontal plane. A1–A4 denote the stationary ultrasonic beacons arranged as an elevated rectangular plane above the robot-arm workspace. The sensor node is mounted above the wrist joint to preserve LoS, while the wrist joint is kept fixed so the sensor-to-end-effector offset remains constant.

The 3D robot-arm experiments were designed to isolate spatial tracking behavior under highly repeatable motion conditions. The mobile sensing node was mounted above the UR10 wrist joint to preserve LoS visibility to the stationary ultrasonic beacons while maintaining a fixed rigid transform between the sensing node and the robot end-effector. During all experiments, the wrist joint remained fixed such that the sensor-to-end-effector offset could be treated as constant throughout the programmed trajectories. Compared with the AGV experiments, the UR10 setup provides a more controlled and repeatable evaluation environment for analyzing vertical-axis behavior, spatial jitter, and trajectory continuity in three-dimensional motion. The repeatability of the robotic path also makes the setup suitable for direct comparison between raw ultrasonic positioning, sliding-window rejection, linear Kalman smoothing, and embedded IMU-ESKF fusion under identical motion conditions. Figure 8 shows the programmed Cartesian reference trajectory used during the 3D evaluation. The selected path contains repeated directional changes and varying spatial geometry in order to expose height-dependent acoustic instability and local beacon-geometry effects.

Table 8 summarizes the fixed metadata used to maintain comparability between the 2D AGV and 3D UR10 evaluations, including the number of repeated trials, nominal motion speed, reference update rate, ultrasonic update range, and IMU sampling frequency. Specifically, the AGV operated at speeds of up to 0.35 m/s, with its mobile sensing node mounted at a height of 1.3 m and navigating beneath stationary beacons elevated at 2.05 m. Similarly, the UR10 arm followed a controlled 0.025 m/s trajectory beneath an elevated reference-beacon plane installed at a height of 2.300 m. Both experimental platforms were evaluated repeatedly under identical estimator settings and comparable synchronization procedures. The repeated-trial design was selected to reduce sensitivity to isolated acoustic events and to allow for statistical comparison between raw ultrasonic localization, sliding-window filtering, and embedded IMU-ESKF mitigation.

The AGV and UR10 experiments intentionally emphasize different aspects of the localization problem. The AGV setup focuses primarily on practical industrial failure conditions such as crossover transitions, acoustic dropout, and intermittent visibility during mobile operation. The UR10 setup focuses primarily on repeatable spatial tracking behavior and controlled analysis of three-dimensional acoustic instability. Together, the two scenarios provide complementary evaluation conditions for the proposed embedded localization architecture.

### 4.2. Evaluation Workflow

Each experiment produces three categories of data. The first category is the raw ultrasonic coordinate stream, including accepted and rejected packets after embedded parsing and CRC validation. The second category is the estimator stream, consisting of prediction-only intervals and position-corrected intervals generated by the embedded sliding-window and IMU-ESKF pipeline. The third category is the independent robotic reference trajectory. For the AGV experiments, the reference is obtained from robot-controller position polling, while for the UR10 experiments, the reference trajectory is reconstructed from commanded node positions and timestamps. These data streams are not assumed to share a common coordinate frame, origin, or sampling grid.

The evaluation procedure first performs temporal alignment between the embedded localization records and the corresponding robotic reference. For the AGV experiments, the planar trajectory is matched against the registered ultrasonic or fused trajectory after timestamp synchronization. For the UR10 trials, the programmed end-effector path is interpolated to the localization timestamps before registration. Because the sensing node was mounted above the wrist joint while the wrist orientation remained fixed throughout the experiment, the sensor trajectory differs from the robot end-effector trajectory by a constant rigid offset. After temporal matching, a rigid registration transform is estimated using the SVD-based Umeyama alignment procedure described in Section 3.4. The resulting transformation is then applied to the complete ultrasonic or fused trajectory. This step removes constant coordinate-frame differences and mounting offsets so that the reported localization error reflects tracking behavior rather than arbitrary frame placement or installation geometry. The evaluation intentionally separates embedded estimator execution from offline quantitative comparison. The robotic references are used exclusively for post-experimental analysis and do not influence the embedded prediction, gating, or correction stages during runtime operation. This separation is important because it ensures that the reported localization performance corresponds to the actual embedded estimator behavior rather than to an externally corrected or smoothed trajectory. The RMSE, peak-error, and CDF results reported below are obtained from firmware-consistent replay of recorded datasets using the same estimator logic implemented in the ESP32-S3 firmware; therefore, they should be interpreted as runtime-equivalent replay results rather than as closed-loop online robot-navigation results. All offline statistical analyses, trajectory alignment, and plotting procedures were executed on a laboratory workstation equipped with an AMD Ryzen 7 7840H CPU and 32 GB of RAM, running a Linux amd64 NixOS distribution and utilizing the Python 3.13 computational environment.

The primary evaluation metrics are trial-mean RMSE, peak error during failure intervals, and recovery behavior after valid acoustic positions return. Trial-mean RMSE summarizes overall positioning accuracy across repeated trajectories, but it can mask severe short-duration localization failures that are particularly relevant for industrial robotic operation. For this reason, peak-error analysis is evaluated specifically during NLoS intervals and crossover transitions, where transient localization discontinuities may affect AGV safety, docking reliability, or trajectory continuity. Recovery behavior is evaluated by analyzing how rapidly the fused trajectory returns toward the robotic reference after acoustic reacquisition and whether the correction introduces large discontinuities into the estimated trajectory. This aspect is especially important for intermittent ultrasonic positioning because abrupt reacquisition corrections can, themselves, produce unstable robot behavior, even if the long-term RMSE remains moderate. To quantify trial-to-trial repeatability, RMSE was calculated independently for each of the ten runs and for each processing method. The reported standard deviation of the sample uses n−1 degrees of freedom, and the 95% confidence interval of the mean is calculated as $\bar{x}\pm t_{0.975,9}s/\sqrt{10}$, with $t_{0.975,9}=2.262$.

The embedded IMU-ESKF architecture is expected to improve primarily the last continuity-related metrics rather than only the average position error. In particular, the inertial prediction stage allows the estimator to maintain a physically plausible motion estimate during missing or rejected acoustic intervals, while validated ultrasonic measurements continue to constrain long-term drift whenever acoustic visibility recovers.

All reported quantitative results are obtained using identical estimator parameters, gating thresholds, and synchronization procedures across repeated trials. This consistent evaluation methodology allows for direct comparison between raw ultrasonic positioning, sliding-window filtering, linear Kalman smoothing, and embedded IMU-ESKF fusion under equivalent experimental conditions.

### 4.3. Baseline Ultrasonic Vulnerabilities

The raw ultrasonic data confirmed the expected sensitivity of acoustic localization to changing environmental visibility and beacon geometry. In 2D AGV experiments, the largest deviations occurred near crossover transitions and partially occluded regions, where the acoustic geometry changed abruptly and beacon visibility became temporarily unstable. In the 3D UR10 experiments, the dominant residual effects appeared as high-frequency spatial oscillations and increased vertical-axis fluctuations. These behaviors are consistent with multipath propagation, local beacon-geometry sensitivity, and intermittent degradation of direct acoustic paths.

**Figure 9:**
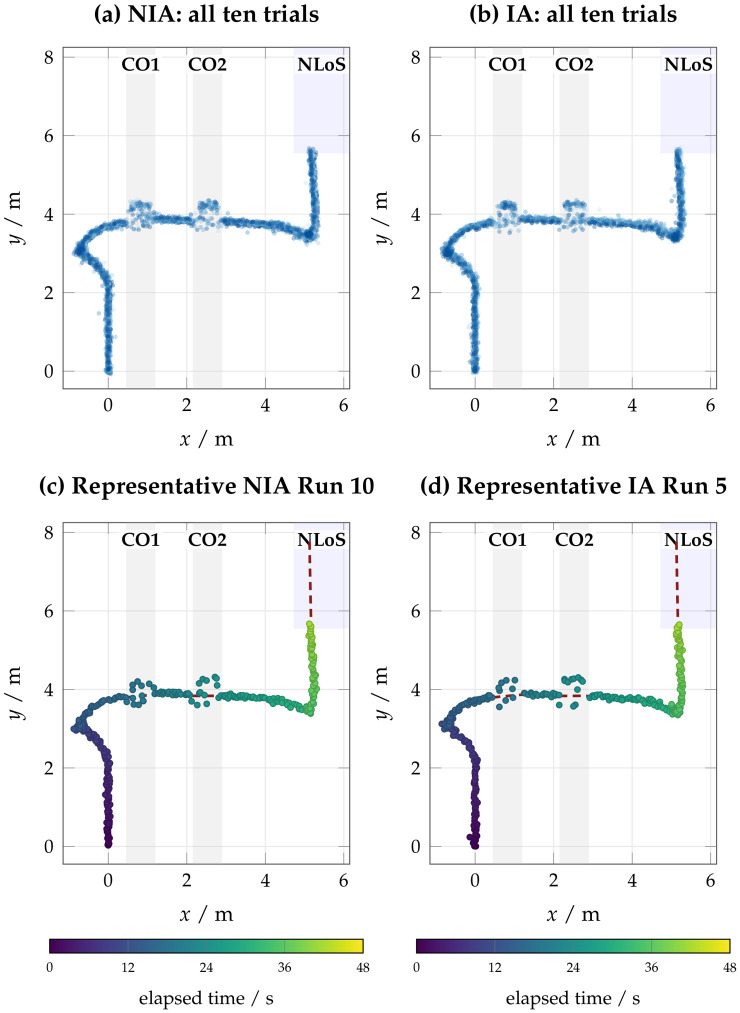
Raw ultrasonic trajectories for the repeated 2D AGV experiments. Panels (**a**,**b**) combine all ten NIA and IA trials using low-opacity, run-specific shades. Panels (**c**,**d**) show representative NIA Run 10 and IA Run 5, selected near the median RMSE of their respective scenarios; sample color denotes elapsed time, and the dark-red dashed line is the AGV reference path. Gray bands mark the two crossover zones (CO1 and CO2), and the blue region marks the NLoS zone. The upper panels show inter-trial spread, whereas the lower panels preserve the temporal progression of individual crossover transitions.

Figure 9 supplements this baseline analysis by showing the raw 2D AGV ultrasonic measurements before IMU-aided correction. Panels (a) and (b) combine all ten trials using low-opacity, run-specific shades to reveal the repeated spatial distribution without forming an opaque point cloud. Panels (c) and (d) show representative trials selected near the median RMSE of their respective scenarios (NIA Run 10 and IA Run 5). The representative ultrasonic samples are color-coded by elapsed time and compared with the AGV reference path, allowing the temporal order of the two crossover transitions and the subsequent NLoS outage to be inspected directly. Even in nominal LoS regions, the measurements exhibit centimeter-level scatter, local bias drift, and increased spread during stop-and-turn maneuvers. The crossover regions contain reduced acoustic update density, together with visible handover jump structures directed toward adjacent beacon regions. In the designated NLoS region, no valid ultrasonic coordinates are available, illustrating the limitations of purely acoustic positioning during prolonged acoustic outages.

The baseline behavior observed in the AGV experiments is particularly relevant for industrial mobile robotics because short-duration localization discontinuities may affect docking, corridor navigation, or trajectory tracking, even when the average positioning error remains moderate. Therefore, the observed crossover artifacts and temporary acoustic dropouts motivate the use of inertial propagation and plausibility-based measurement rejection within the embedded localization pipeline.

**Figure 10:**
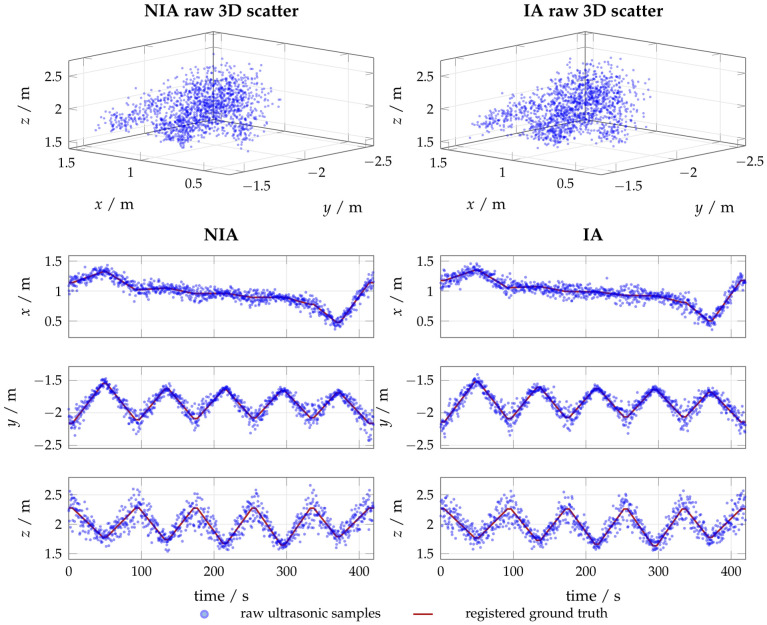
Raw ultrasonic scatter and time-domain coordinate comparison for the 3D UR10 NIA and IA trials. The upper row shows raw 3D ultrasonic samples from all repeated trials combined. The lower rows show one representative run over a single programmed path cycle; blue markers are raw ultrasonic samples, and the red line is the interpolated robot ground truth after visualization registration to the ultrasonic coordinate frame.

Figure 10 extends the same baseline analysis to the 3D UR10 trials. The upper row preserves the raw 3D ultrasonic scatter for all repeated NIA and IA trials, showing the measured spatial envelope without additional post-processing. Direct visualization of the full 3D trajectory, together with the programmed robotic reference, is difficult to interpret visually; therefore, the lower rows additionally separate one representative trajectory into x(t), y(t), and z(t) components. For visualization purposes, the programmed UR10 reference trajectory is interpolated from the path in Figure 8 and rigidly registered to the ultrasonic frame. This registration does not alter the embedded output itself but allows axis-specific acoustic behavior to be inspected consistently for visualization. In particular, the visualization highlights local acoustic jitter, transient deviations, and the stronger vertical-axis instability present before IMU-aided filtering.

Table 9 summarizes the baseline RMSE values obtained before embedded IMU-ESKF fusion. The processing chain is separated into four levels to clarify where the improvement originates. Raw ultrasonic data include all valid acoustic coordinates. Sliding-window-only processing suppresses isolated acoustic outliers and handover jumps before any temporal smoothing is applied. Adding the no-IMU LKF baseline further smooths short-duration fluctuations but still relies primarily on acoustic measurements, together with constant-velocity extrapolation during missing-update intervals. This limitation becomes particularly visible during prolonged NLoS intervals and crossover transitions, where the localization system temporarily loses reliable acoustic constraints. In these situations, smoothing alone cannot maintain physically plausible trajectory continuity over longer durations. Therefore, the baseline analysis motivates the embedded IMU-aided ESKF architecture, whose primary purpose is not only reducing average positioning noise but also maintaining bounded trajectory behavior during intermittent acoustic degradation and measurement dropout.

### 4.4. Embedded IMU-ESKF Error Mitigation

The embedded mitigation stage was evaluated through firmware-consistent replay of the AGV and UR10 experimental datasets using the same gating and estimator logic implemented on the ESP32-S3 firmware. Valid ultrasonic measurements first pass the sliding-window kinematic gate described in Section 3.5. Accepted measurements are then used as absolute position corrections within the ESKF, while rejected or missing acoustic samples trigger IMU-only prediction. This runtime-equivalent replay used the identical gating, propagation, and correction logic implemented in the ESP32-S3 firmware; it was not an offline trajectory optimization or smoothing procedure. The comparison explicitly separates the effect of the acoustic gate, the no-IMU LKF smoother, and the IMU-aided ESKF. The sliding-window gate removes physically implausible acoustic samples before state correction. The sliding window-plus-LKF baseline is treated as the no-IMU kinematic control: it smooths the remaining valid acoustic measurements and can bridge only very short update gaps through constant-velocity extrapolation, but it has no independent inertial information during NLoS intervals. In contrast, the ESKF maintains a continuously propagated motion state using measured acceleration and angular-rate information and only re-introduces ultrasonic corrections when the incoming measurements are considered physically plausible by the embedded gate.

The mitigation effect is strongest in the two dominant failure modes identified in Figure 9 and Figure 10. In the AGV experiments, crossover regions contain sparse acoustic updates, together with visible handover jumps, while the NLoS region contains intervals without valid ultrasonic measurements. During these intervals, the ESKF rejects implausible crossover outliers and continues propagating the state estimate using IMU prediction. In the UR10 experiments, the dominant residual effect appears as height-dependent 3D fluctuations, especially in the vertical component. Here, the ESKF reduces high-frequency spatial oscillations by using inertial propagation as a local motion prior and by preventing large acoustic residuals from immediately perturbing the nominal state estimate. Table 10 summarizes the peak-error mitigation obtained with the embedded IMU-ESKF pipeline.

The failure intervals are not limited to isolated outlier samples. Therefore, Table 11 reports a compact continuity metric for the 2D AGV trials. Across the entire executed trajectory, approximately 78% of the positioning time occurred under favorable LoS conditions, while the remaining 22% traversed degraded acoustic environments (approximately 13% NLoS occlusion and 9% crossover transitions). The NLoS prediction interval is computed according to the recorded zone labels and denotes the duration over which ultrasonic updates are largely unavailable or fail validation. The acoustic dropout rates are computed according to the recorded ultrasonic packet stream. These values quantify the acoustic degradation that the firmware-consistent ESKF replay must bridge without relying on offline smoothing.

The results indicate that the ESKF primarily reduces the tail of the error distribution rather than only improving already stable LoS positioning. In the AGV experiments, peak errors caused by crossover transitions and turn-related acoustic scatter are reduced from approximately 527–538 mm to 181–193 mm after embedded ESKF fusion, while the estimator remains continuous through NLoS prediction intervals of approximately 6–7 s, as shown in Table 11. For the UR10 experiments, the maximum 3D error remains larger because the trajectories intentionally include height-dependent geometric sensitivity, but the ESKF still reduces the peak error by approximately 59–60%. This behavior is consistent with the intended role of the inertial subsystem. The IMU does not replace ultrasonic absolute positioning as the global position reference; instead, it stabilizes the estimated trajectory during temporary acoustic failures and prevents short-duration positioning failure from producing large discontinuities in the embedded state estimate.

The positioning statistics in Table 12 show the same overall trend and separate the contribution of each processing layer. Sliding-window-only gating provides the first major improvement by removing large acoustic outliers, reducing 2D AGV trial-mean RMSE from 101.2–104.1 mm to 62.4–65.1 mm and 3D UR10 trial-mean RMSE from 157.6–158.4 mm to 126.9–128.6 mm. Adding the no-IMU LKF baseline further reduces short-term fluctuation, especially in the 3D experiments, and represents the control case for acoustic filtering plus kinematic smoothing without real inertial propagation. The embedded IMU-ESKF logic provides the largest additional improvement during periods of degraded acoustic visibility, reducing trial-mean RMSE to 47.2–48.7 mm in 2D and 80.2–80.5 mm in 3D. This comparison indicates that the reported improvement cannot be attributed solely to outlier rejection or generic smoothing: the no-IMU LKF baseline already captures those effects, while the additional ESKF improvement is associated with measured inertial propagation during rejected or missing acoustic updates. The trial-level standard deviations and confidence intervals preserve the same method ordering in every scenario and show that the improvement is not caused by one isolated run. Therefore, the IA and NIA datasets are interpreted as complementary validation scenarios for the proposed mitigation pipeline. Under the investigated laboratory conditions, the two configurations employed comparable beacon placement, identical trajectories, the same estimator parameters, and a common synchronization procedure. The observed similarity in localization performance indicates that the dominant error sources were associated with crossover transitions, acoustic outages, and local beacon-geometry effects rather than with the underlying acoustic transmission topology. Nevertheless, ten trials provide an estimate of repeatability under these controlled conditions rather than broad population-level validation across different buildings, beacon layouts, or robotic platforms.

To assess the robustness of the proposed approach with respect to parameter selection, a local one-factor-at-a-time sensitivity analysis was conducted around the nominal parameter set defined in Table 6. For each analysis, a single parameter was varied while all remaining estimator, filtering, and synchronization parameters were held constant. Table 13 summarizes the resulting replay-based ESKF RMSE values and peak failure-interval errors, averaged across the two AGV scenarios and the two UR10 scenarios. Within each table entry, the reported results correspond to the sequence of parameter values listed in the second column. The investigated variations include stricter and more permissive outlier-rejection thresholds, shorter and longer sliding-window lengths, and lower and higher ultrasonic measurement covariance values. Across the examined parameter ranges, moderate changes in the parameter settings resulted in only limited variations of the absolute error metrics. More importantly, the relative performance ranking of the evaluated localization approaches remained unchanged compared with the results reported in Table 12. These findings indicate that the reported performance improvements are robust to moderate parameter variations and do not depend on a narrowly tuned set of threshold or covariance values.

**Figure 11:**
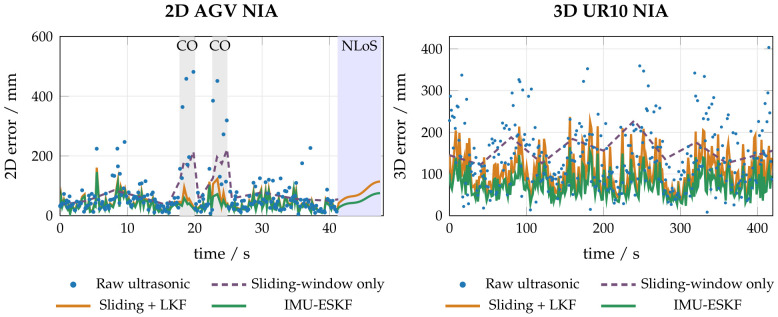
Representative firmware-consistent replay error histories for one AGV NIA run and one UR10 NIA path cycle. The AGV panel includes two crossover zones (abbreviated as CO in the plot) and one NLoS interval; the UR10 panel shows the corresponding runtime-equivalent 3D replay over one programmed path cycle. Raw ultrasonic errors are shown as blue markers, sliding-window-only gating as purple dashed curves, sliding-window LKF output as the no-IMU kinematic baseline in orange, and IMU-ESKF output as green curves.

Figure 11 illustrates why the numerical improvement is dominated mainly by degraded acoustic intervals rather than by already well-behaved LoS segments. During stable AGV motion under good acoustic visibility, the raw ultrasonic positioning error is already comparatively small, and the ESKF behaves primarily as a bounded correction mechanism. During crossover transitions and NLoS intervals, however, the acoustic measurements either become sparse, exhibit abrupt jumps, or disappear completely. Under these conditions, the embedded ESKF maintains bounded trajectory behavior by switching to inertial prediction and delaying acoustic correction until plausible measurements return. This behavior is especially important for practical robotic operation because abrupt reacquisition jumps can be more problematic for navigation stability than moderate steady-state noise. The same effect can also be observed in the UR10 experiments. The ESKF substantially reduces high-amplitude 3D fluctuations while preserving the repeated stop-and-move structure of the programmed robot trajectory. Therefore, the inertial propagation acts primarily as a continuity-preserving mechanism during intermittent acoustic degradation rather than as a replacement for absolute positioning.

**Figure 12:**
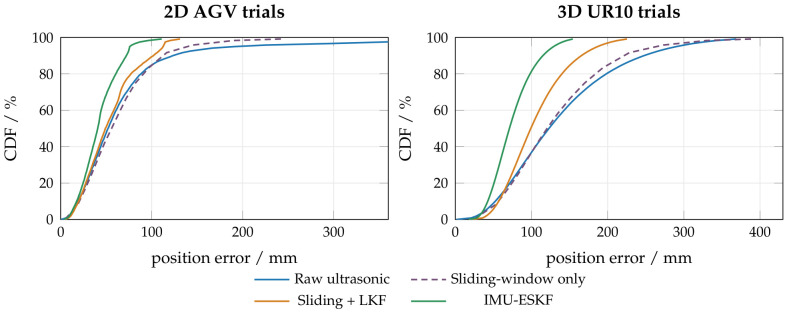
Cumulative error distributions for all repeated trials combined. The left panel combines all 2D AGV NIA and IA runs; the right panel combines all 3D UR10 NIA and IA runs. The ESKF curves shift toward lower error and shorten the high-error tail compared with raw ultrasonic data, sliding-window-only gating, and the sliding window-plus-LKF no-IMU kinematic baseline.

Figure 12 provides a compact statistical view of the resulting tail-error reduction through cumulative distribution functions (CDFs). In both the 2D and 3D experiments, the ESKF curves rise earlier and reach high cumulative probability at lower positioning errors compared with raw ultrasonic positioning, sliding-window-only gating, and the no-IMU LKF baseline. This representation is particularly informative because the most safety-relevant localization failures are not the central LoS samples but the comparatively rare large deviations caused by crossover jumps, acoustic dropout, or height-sensitive three-dimensional geometry. Therefore, the CDF analysis indicates that the embedded IMU-ESKF logic improves not only average RMSE but also replay-based robustness during intermittent acoustic degradation.

### 4.5. Embedded Runtime Performance

In addition to localization accuracy, the embedded implementation must be evaluated as a real-time system. An estimator that improves RMSE only during offline analysis is insufficient for AGV or robotic deployment if it cannot maintain the required IMU update rate, if ultrasonic packets are dropped during communication bursts, or if diagnostic network traffic interferes with estimator timing. Therefore, the ESP32-S3 implementation requires a separate runtime characterization in addition to the positioning analysis presented in the previous Section 3.1. The most relevant runtime quantities are the estimator execution time, effective IMU sampling rate, accepted ultrasonic update rate, UART packet rejection rate, queue occupancy, WebSocket output rate, and remaining system memory. These quantities determine whether the embedded localization architecture can sustain continuous operation while simultaneously handling inertial propagation, ultrasonic parsing, outlier rejection, and diagnostic communication.

Table 14 reports firmware-consistent runtime values for the ESP32-S3-WROOM-1 implementation used in this study. The measurements correspond to the implemented dual-core task structure, interrupt-driven ICM-20948 acquisition, 500 kbaud Marvelmind UART parser, and 60 Hz diagnostic WebSocket stream described in Section 3.1.

The runtime measurements indicate that the embedded architecture can sustain the required IMU and ultrasonic processing rates without observable estimator instability. The interrupt-driven IMU acquisition maintained an effective rate of approximately 499.6 Hz, closely matching the nominal 500 Hz configuration. The mean ESKF prediction step required 0.74±0.12 ms, while ultrasonic correction updates required approximately 1.36±0.21 ms when valid acoustic measurements were available. Both values remain well below the corresponding update intervals, leaving timing margin for communication and diagnostic tasks.

The accepted ultrasonic update rate remained within the expected 5–15 Hz range, with measured variation primarily determined by acoustic visibility and beacon geometry rather than by parser limitations. The UART CRC rejection rate remained low, at 0.07%, indicating stable packet framing and reliable serial communication throughout the experiments.

The sliding-window rejection rate varied significantly between nominal LoS operation and degraded acoustic conditions. During stable LoS intervals, only approximately 3.1% of ultrasonic samples were rejected, whereas crossover and NLoS intervals produced rejection rates approaching 24.8%. This behavior is consistent with the intended role of the kinematic gate as a lightweight measurement-quality classifier under changing acoustic conditions.

Importantly, no estimator queue overflows were observed during the experiments. Therefore, the bounded synchronous communication channel between the UART parser and estimator task remained stable, even during burst-like packet arrival. Likewise, the available heap memory remained above 124 kB throughout runtime operation despite concurrent Wi-Fi access-point management, WebSocket streaming, and estimator execution.

Runtime characterization was performed with the browser-based WebSocket diagnostic stream enabled, matching the monitoring configuration used during experimental data acquisition. This represents a comparatively demanding runtime scenario because network communication, visualization support, and estimator execution operate concurrently on the embedded platform.

For practical industrial deployment, the same fused localization state could alternatively be exposed through a deterministic robot-control interface, while the WebUI remains primarily a diagnostic and logging mechanism. Therefore, the presented runtime results indicate that the ESP32-S3 architecture is capable of supporting continuous embedded ultrasonic-inertial localization, together with concurrent monitoring and data export without compromising estimator timing stability.

## 5. Discussion

The results support the central premise of this work: ultrasonic indoor positioning in laboratory environments should be treated as an embedded robustness and sensor-fusion problem rather than as a purely acoustic coordinate-generation problem. The raw ultrasonic measurements provide absolute geometric information when direct acoustic paths are available, but the experiments in Figure 9 and Figure 10 demonstrate that this information is not uniformly reliable under realistic operating conditions. In the 2D AGV experiments, the dominant failure modes are sparse updates, crossover handover jumps, and complete acoustic dropouts in NLoS regions. In the 3D UR10 experiments, the dominant effects appear as increased spatial scatter and height-dependent fluctuations. Although these effects differ visually, they share the same localization implication: the estimator must distinguish between trustworthy and corrupted acoustic measurements while maintaining a physically plausible trajectory during periods of degraded visibility.

Therefore, a key contribution of this work is not only the reduction in average positioning error but the explicit mitigation of intermittent acoustic failures within an embedded runtime architecture. The proposed pipeline combines packet validation, kinematic plausibility gating, and inertial prediction directly on the ESP32-S3 sensing node, allowing the estimator to maintain continuity during missing or corrupted acoustic updates without relying on a host-side processing computer. In this respect, the presented approach differs from purely post-processing-based smoothing methods and nominal-condition localization studies by focusing specifically on embedded robustness under degraded acoustic visibility.

The quantitative replay results show that the strongest improvements appear in the high-error tail rather than in already stable LoS samples. In the AGV experiments, the firmware-consistent IMU-ESKF replay reduces trial-mean RMSE from 101.2–104.1 mm for raw ultrasonic positioning data to 47.2–48.7 mm, while peak failure-interval errors decrease from approximately 527–538 mm to 181–193 mm. In the 3D UR10 experiments, where the trajectories intentionally include stronger height-dependent uncertainty, trial-mean RMSE decreases from 157.6–158.4 mm to 80.2–80.5 mm, and peak-error reductions reach approximately 59–60%. These improvements are particularly relevant because short-duration localization failures can be more critical for AGV navigation, docking, and robotic path continuity than moderate steady-state positioning noise.

The experiments also show that inertial fusion alone is insufficient without explicit measurement validation. The sliding-window gate remains necessary, even in the presence of an ESKF, because reflected-path measurements or crossover jumps may otherwise enter the estimator as valid absolute corrections and pull the nominal state away from the physical trajectory. Therefore, the gating stage acts as a deterministic plausibility layer before probabilistic sensor fusion. This layered approach is especially important during crossover intervals, where valid and invalid acoustic measurements may occur close together in time.

Compared with the sliding window-plus-LKF no-IMU baseline, the embedded IMU-ESKF contributes continuous motion propagation during periods of degraded or unavailable ultrasonic measurements. While the LKF can smooth measurements and bridge short gaps through kinematic extrapolation, the ESKF maintains a physically consistent state estimate based on measured inertial information. Consequently, the comparison between the LKF and IMU-ESKF configurations serves to distinguish the effects of acoustic outlier rejection and trajectory smoothing from those of inertial propagation. This distinction is particularly relevant for AGV stop-and-go motion, docking maneuvers, directional changes, and robot-arm trajectories, where constant-velocity assumptions are only locally valid and prediction accuracy increasingly depends on measured platform dynamics.

Another important contribution of the work is the embedded system integration itself. The proposed architecture moves acquisition, packet validation, outlier rejection, inertial propagation, and estimator execution directly onto the ESP32-S3-WROOM-1 platform. This reduces dependence on external computation during runtime operation and allows the localization node to operate as a compact, self-contained sensing unit. The browser-based WebUI remains useful for diagnostics, visualization, and CSV export, but the localization pipeline itself no longer depends on a continuously connected host computer.

The runtime characterization further indicates that the selected architecture is compatible with the required IMU and ultrasonic update rates. The dual-core task organization allows communication-oriented services and estimator execution to remain separated, while the interrupt-driven IMU acquisition and bounded communication queues maintain deterministic estimator timing, even during simultaneous WebSocket monitoring.

The layered failure-handling structure also provides practical diagnostic advantages. CRC validation removes malformed serial packets, the sliding-window gate rejects kinematically implausible acoustic coordinates, and the ESKF bridges missing measurements through inertial prediction. Separating these functions simplifies both tuning and troubleshooting because communication failures, acoustic outliers, and prolonged visibility loss can be analyzed independently rather than appearing as a single undifferentiated localization error source.

Several limitations should be considered when interpreting the reported results. The experimental evaluation was conducted in a single laboratory environment using one beacon-layout configuration, one family of AGV trajectories, and one family of UR10 trajectories, without the presence of human operators or other dynamic obstacles. Consequently, the findings should be regarded as a controlled laboratory validation of the proposed embedded ultrasonic–inertial mitigation framework rather than as evidence of performance under diverse industrial operating conditions. The inertial subsystem requires careful calibration and consistent frame alignment; otherwise, gyroscope bias and gravity misalignment may accumulate as false motion during acoustic outages. The ESKF covariance matrices and gating thresholds are also scenario-dependent because acoustic variance changes with beacon geometry, carrier height, and local occlusion conditions. Over long NLoS intervals, inertial drift will inevitably accumulate, meaning that sensor fusion reduces but does not eliminate the need for careful beacon placement and acoustic service-area design. In addition, the ten repeated trials quantify short-term repeatability within the tested laboratory configurations, but they are not sufficient for unrestricted statistical generalization to substantially different environments.

Accordingly, the reported localization errors should be interpreted relative to repeatable robotic reference trajectories with known manufacturer-specified uncertainty rather than as absolute positioning errors obtained from an independent metrological tracking system. The present IA/NIA comparison is limited to localization performance under the investigated laboratory conditions and should not be interpreted as a comprehensive communication-layer evaluation. Metrics such as the packet reception rate, packet loss rate, visible-beacon count, latency, handover behavior, scalability, and recovery performance were outside the scope of the present experiments and would require dedicated Marvelmind diagnostic logging, together with controlled network-load testing. Therefore, future validation should include additional deployment sites; alternative beacon geometries; independently repeated experimental campaigns; dynamic obstacles; human operators within the workspace; extended-duration operation; detailed IA/NIA communication diagnostics; and—where available—an independent, high-precision reference system such as an optical motion-capture system, laser tracker, or total station.

The presented mitigation results are based on firmware-consistent replay of the embedded estimator logic using the recorded AGV and UR10 datasets. Although this methodology preserves the runtime estimator structure and timing assumptions, longer continuous deployments would provide additional insight into long-term drift behavior, queue stability, recovery dynamics after prolonged acoustic outages, and deterministic robot-control integration under extended operation.

## 6. Conclusions

This paper evaluated ultrasonic indoor positioning for AGV and robot-arm applications and presented an embedded ultrasonic–inertial mitigation architecture implemented on an ESP32-S3-WROOM-1 platform. The experiments confirm that Marvelmind ultrasonic positioning can provide accurate coordinates under favorable LoS conditions but that the raw acoustic output remains vulnerable to multipath propagation, crossover handover jumps, intermittent update loss, NLoS occlusion, and height-dependent 3D scatter.

The main contribution of this work is the integration of embedded packet validation, kinematic outlier rejection, high-rate IMU acquisition, and a 15-state error-state Kalman filter into a unified runtime localization pipeline designed specifically for degraded acoustic conditions. Rather than focusing solely on nominal positioning accuracy, the proposed architecture targets continuity preservation and robustness during intermittent or corrupted ultrasonic visibility.

Across the evaluated AGV and UR10 datasets, firmware-consistent replay of the embedded IMU-ESKF logic substantially reduced both trial-mean RMSE and failure-interval divergence. Relative to raw ultrasonic positioning, replay-based trial-mean RMSE was reduced to 47.2–48.7 mm in the 2D AGV experiments and to 80.2–80.5 mm in the 3D UR10 experiments. Peak failure-interval errors were reduced by 64.2–65.7% in the 2D crossover/NLoS scenarios, and peak height-sensitive 3D errors were reduced by 58.8–60.0% in the UR10 scenarios. Under the tested conditions, the NIA and IA configurations exhibited similar localization behavior and are therefore interpreted as parallel robustness assessments of the proposed embedded mitigation framework rather than as evidence for architecture-level superiority.

The results indicate that integrating inertial propagation directly within the embedded ultrasonic sensing node can substantially improve localization robustness during periods of degraded or intermittent acoustic measurements in controlled laboratory AGV and robot-arm applications. Broader industrial deployment remains a topic for future work and requires validation under varying beacon geometries, dynamic occlusions, human presence, and long-duration operation.

Future work will focus on longer continuous deployments, alternative beacon layouts, moving obstacles, human presence in the workspace, hardware-timestamped latency analysis, and deterministic robot-control interfaces in addition to the current diagnostic WebUI. Specifically, adaptive tuning of the ultrasonic measurement covariance for different acoustic conditions (such as dynamically switching between stable LoS and degraded NLoS parameters) will be investigated. To mitigate inevitable inertial drift during prolonged NLoS intervals, future extensions will also explore the integration of visual positioning assistance, such as visual–inertial odometry (VIO) or fiducial markers (e.g., AprilTags) [37,38]. These extensions are required before generalizing the present controlled-laboratory findings to dynamic industrial production environments.

## Figures and Tables

**Table 1:** Failure-mode overview for ultrasonic indoor positioning and corresponding mitigation strategy.

Failure Mode	Cause	Observed Effect	Mitigation
Multipath	Reflections from walls, equipment, or carrier structures	Local scatter, biased coordinates, and short trajectory bending	Sliding-window gate rejects kinematically implausible acoustic samples before fusion
Crossover	Transition between adjacent beacon service regions	Handover jumps, reduced update density, and short dropouts	ESKF prediction maintains trajectory continuity while jump samples are rejected
NLoS	Direct acoustic path blocked by robot body, obstacles, or workspace furniture	Missing ultrasonic updates or sparse reacquisition samples	IMU propagation bridges acoustic gaps until validated ultrasonic updates return
3D geometry degradation	Weak vertical geometry and height-dependent beacon visibility	Increased vertical jitter and larger 3D error tail	Lever-arm-aware ESKF correction and inertial prediction reduce spatial fluctuation

**Table 2:** Principal technical specifications of the ESP32-S3-WROOM-1 embedded microcontroller [28].

Parameter	Specification
Microprocessor Core	Xtensa dual-core 32-bit LX7
Maximum Clock Frequency	240 MHz
Internal SRAM	512 KB
Integrated PSRAM	8 MB
Integrated Flash	8 MB
Wireless Connectivity	2.4 GHz Wi-Fi & Bluetooth 5 (LE)
Peripheral Interfaces	UART, I2C, SPI, USB OTG
Operating Voltage	3.0 V–3.6 V

**Table 3:** Hardware specifications and signal conditions of the Marvelmind Super-Beacon serving reference stations [22].

Parameter	Specification
Recommended operating range	Up to 30 m (line of sight)
Maximum tracking distance	Up to 50 m (1D in laboratory conditions)
Ultrasonic frequencies	19, 22, 25, 28, 31, 34, 37, or 45 kHz
Radio frequency (synchronization)	915 MHz (US/ISM) or 868 MHz (EU/SRD)
Radio output power	<10 mW (<10 dBm)
Power supply	5V Micro-USB or internal LiPo battery
Nominal precision	±2 cm (differential)

**Table 4:** Embedded implementation used in the experiments.

Component	Implementation
Controller	ESP32-S3-WROOM-1 running Rust 2021 with ESP-IDF
IMU acquisition	ICM-20948 on I2C0, 400 kHz bus, 500 Hz data-ready interrupt
Ultrasonic input	Marvelmind UART2 stream, 500 kbaud, CRC16 packet validation
Fusion core	15-state ESKF in single precision with quaternion attitude and IMU bias states
Measurement model	Ultrasonic position update with configurable lever arm between IMU and acoustic reference point
Communication	Local Wi-Fi access point, embedded HTTP server, WebSocket binary stream at 60 Hz
Task layout	Marvelmind task on Core 0, estimator task on Core 1, Wi-Fi and HTTP services on Core 0

**Table 5:** Hardware and mounting parameters used in the ESP32-S3 ultrasonic–inertial experiments.

Item	Value Used in This Work	Notes
Microcontroller	ESP32-S3-WROOM-1	Embedded Rust/ESP-IDF acquisition and fusion node
IMU	ICM-20948	Accelerometer/gyroscope sampled from data-ready interrupt
IMU sampling	500 Hz on I2C0, 400 kHz bus	200 static samples used for initial bias and gravity-alignment calibration
Ultrasonic system	Marvelmind Super-Beacon	Evaluated in NIA and IA configurations
Ultrasonic packet input	UART2, 500 kbaud, CRC16 validation	Coordinates converted from millimeters to meters in firmware
Diagnostic interface	Wi-Fi AP, HTTP server, WebSocket stream	Browser WebUI visualization and CSV export at 60 Hz
Sensor-node lever arm	$l={\left[-0.0275,\;0,\;0\right]}^{T}$ m	Body-frame offset from IMU origin to ultrasonic acoustic reference point
Carrier mounting	AGV top plate; UR10 wrist-joint fixture	UR10 wrist joint kept fixed to preserve a constant sensor-to-end-effector transform

**Table 6:** ESKF and outlier-gating parameters used for the ESP32-S3 evaluation and initial tuning.

Parameter	Symbol/Code Field	Unit	Final Value
Accelerometer white noise	acc_noise	m s^−2^/$\sqrt{\mathrm{Hz}}$	0.020
Gyroscope white noise	gyro_noise	rad s^−1^/$\sqrt{\mathrm{Hz}}$	0.005
Accelerometer bias random walk	acc_bias_walk	m s^−3^/$\sqrt{\mathrm{Hz}}$	$1.0\times 10^{-4}$
Gyroscope bias random walk	gyro_bias_walk	rad s^−2^/$\sqrt{\mathrm{Hz}}$	$1.0\times 10^{-5}$
Ultrasonic measurement standard deviation	ultrasound_std	m	0.030
Sliding-window length	*N*	samples	10
Velocity rejection threshold	$v_{\max}$	m s^−1^	1.0
Acceleration rejection threshold	$a_{\max}$	m s^−2^	2.0
Sliding-window Z-score threshold	$z_{\max}$	–	3.0
Lever arm	l	m	${\left[-0.0275,\;0,\;0\right]}^{T}$

**Table 7:** Experimental scenarios and reference systems.

Scenario	Dimension	Architecture	Carrier	Ground Truth
1	2D	NIA	AGV	Robot position polling
2	2D	IA	AGV	Robot position polling
3	3D	NIA	UR10 arm	Interpolated arm path
4	3D	IA	UR10 arm	Interpolated arm path

**Table 8:** Experimental metadata for reproducible evaluation.

Metadata Item	AGV 2D	UR10 3D
Number of repeated trials	10	10
Nominal speed profile	0–0.35 m/s	0.025 m/s
Beacon coordinates	As shown in Figure 7	As shown in Figure 8
Ground-truth update rate	10 Hz	10 Hz
Ultrasonic update rate	5–15 Hz	3–15 Hz
IMU sample rate	500 Hz	500 Hz

**Table 9:** Baseline ultrasonic performance before IMU-ESKF fusion. Values are trial-mean RMSE across ten trials in millimeters.

Scenario	Dimension	Arch.	Raw Ultrasonic	Sliding Only	Sliding + LKF
1	2D	NIA	104.1	65.1	62.6
2	2D	IA	101.2	62.4	61.3
3	3D	NIA	158.4	128.6	114.1
4	3D	IA	157.6	126.9	113.6

**Table 10:** Peak-error mitigation in the firmware-consistent replay data. Values are in millimeters; gain is the peak-error reduction from raw ultrasonic data to IMU-ESKF output. CO denotes crossover intervals.

Scenario	Failure Mode	Raw Peak	Sliding Peak	No-IMU LKF Peak	IMU-ESKF Peak	Gain
2D NIA	CO/NLoS	538.2	228.4	211.6	192.8	64.2%
2D IA	CO/NLoS	526.8	240.2	198.5	180.8	65.7%
3D NIA	3D degradation	495.7	433.5	296.1	204.3	58.8%
3D IA	3D degradation	499.3	470.9	289.4	199.8	60.0%

**Table 11:** Failure-interval continuity metrics for the 2D AGV trials. Values are averaged over ten repeated runs per scenario.

Scenario	Mean NLoS Prediction Interval	NLoS Acoustic Dropout	Crossover Acoustic Dropout	ESKF Peak Failure Error
2D NIA	6.24 s	94.6%	37.7%	192.8 mm
2D IA	6.51 s	93.8%	39.2%	180.8 mm

**Table 12:** Statistical positioning accuracy and trial-level repeatability after firmware-consistent replay. Mean error, median, 95th percentile, and maximum are pooled-sample descriptive statistics of the Euclidean localization error. Trial RMSE is reported across ten independent trials as mean ± sample SD, followed by a separate Student-*t* 95% confidence interval. All values are in millimeters.

Scenario	Method	Mean Error	Median	95th Perc.	Max	RMSE Mean ± SD	95% CI (n=10)
2D NIA	Raw	72.5	53.5	202.6	538.2	104.1±10.8	[96.4,111.8]
2D NIA	Sliding window only	56.1	50.5	118.0	228.4	65.1±6.8	[60.3,69.9]
2D NIA	Sliding + LKF (no IMU)	55.1	50.9	112.5	211.6	62.6±4.0	[59.7,65.5]
2D NIA	ESKF	43.6	41.5	76.0	192.8	48.7±4.1	[45.8,51.6]
2D IA	Raw	69.1	50.3	181.2	526.8	101.2±7.3	[96.0,106.4]
2D IA	Sliding window only	53.3	47.5	117.1	240.2	62.4±4.5	[59.2,65.6]
2D IA	Sliding + LKF (no IMU)	53.6	47.9	112.5	198.5	61.3±2.4	[59.6,63.0]
2D IA	ESKF	42.2	39.8	75.8	180.8	47.2±2.5	[45.4,49.0]
3D NIA	Raw	138.4	123.3	290.6	495.7	158.4±4.0	[155.5,161.3]
3D NIA	Sliding window only	111.8	98.9	236.1	433.5	128.6±3.2	[126.3,130.9]
3D NIA	Sliding + LKF (no IMU)	106.0	99.4	186.8	296.1	114.1±2.4	[112.4,115.8]
3D NIA	ESKF	75.4	71.2	129.0	204.3	80.5±1.6	[79.4,81.6]
3D IA	Raw	137.6	122.4	289.8	499.3	157.6±5.9	[153.4,161.8]
3D IA	Sliding window only	110.2	97.0	234.7	470.9	126.9±4.8	[123.5,130.3]
3D IA	Sliding + LKF (no IMU)	105.5	98.8	186.6	289.4	113.6±3.5	[111.1,116.1]
3D IA	ESKF	75.1	70.8	128.9	199.8	80.2±2.4	[78.5,81.9]

**Table 13:** Local one-factor-at-a-time sensitivity of the firmware-consistent IMU-ESKF replay. RMSE and peak values are in millimeters and are averaged over NIA and IA trials within each motion class.

Parameter	Value	AGV RMSE	UR10 RMSE	AGV Peak	UR10 Peak
Window length (*N*)	5 samples	50.1	83.9	192.8	208.5
	10 samples	47.9	80.3	186.8	202.0
	15 samples	48.8	81.8	185.3	200.4
	20 samples	49.5	82.9	188.9	204.3
Velocity gate ($v_{\max}$)	0.5 m s^−1^	49.6	83.1	189.1	204.5
	1.0 m s^−1^	47.9	80.3	186.8	202.0
	1.5 m s^−1^	49.1	82.3	191.3	206.9
Acceleration gate ($a_{\max}$)	1.0 m s^−2^	49.3	82.6	186.1	201.2
	2.0 m s^−2^	47.9	80.3	186.8	202.0
	3.0 m s^−2^	49.0	82.1	190.4	205.9
Ultrasonic std. ($\sigma_{us}$)	0.02 m	48.8	81.8	190.6	206.1
	0.03 m	47.9	80.3	186.8	202.0
	0.05 m	49.8	83.4	184.9	200.0
Z-score gate ($z_{\max}$)	2.5	48.5	81.3	185.5	200.6
	3.0	47.9	80.3	186.8	202.0
	3.5	48.9	81.9	190.0	205.5

**Table 14:** Embedded runtime performance for the ESP32-S3-WROOM-1 firmware build used in this study.

Metric	Target/Expected Value	Measured Value	Comment
IMU data-ready rate	500 Hz	499.6±0.8 Hz	ICM-20948 data-ready interrupt
Mean ESKF prediction time	<2 ms	0.74±0.12 ms	15-state f32 prediction step
Mean ultrasonic update time	<5 ms	1.36±0.21 ms	Position update when a valid packet arrives
Accepted ultrasonic packet rate	5–15 Hz	9.8±2.6 Hz	Marvelmind UART at 500 kbaud
UART CRC rejection rate	near 0%	0.07%	Rejected parser frames
Sliding-window rejection rate	Scenario-dependent	3.1% LoS/24.8% crossover	Acoustic outlier load
WebSocket output rate	60 Hz	59.4±1.1 Hz	Diagnostic monitoring stream
Estimator queue overflow count	0	0	16-sample synchronous channel
Minimum free heap	>80 kB	124 kB	Wi-Fi AP and HTTP server active

## Data Availability

The data presented in this study are available on request from the corresponding author due to institutional restrictions and the use of proprietary software and resources belonging to our institute.

## Abbreviations

The following abbreviations are used in this manuscript:

AGV	Automated guided vehicle
AP	Access point
CDF	Cumulative distribution function
CO	Crossover interval
CPU	Central processing unit
CRC	Cyclic redundancy check
CRC16	16-bit cyclic redundancy check
ESKF	Error-state Kalman filter
ESP-IDF	Espressif IoT Development Framework
ESP32-S3	Espressif ESP32-S3 microcontroller family
HTTP	Hypertext Transfer Protocol
I2C	Inter-integrated circuit
IA	Inverse architecture
ICM-20948	Nine-axis inertial measurement unit
IMU	Inertial measurement unit
LKF	Linear Kalman filter
LoS	Line of sight
NIA	Non-inverse architecture
NLoS	Non-line of sight
PSRAM	Pseudo-static random-access memory
RFID	Radio-frequency identification
RMSE	Root mean square error
SVD	Singular value decomposition
ToF	Time of flight
UART	Universal asynchronous receiver–transmitter
UR10	Universal Robots UR10 collaborative robot
